# *Orf165* is associated with cytoplasmic male sterility in pepper

**DOI:** 10.1590/1678-4685-GMB-2021-0030

**Published:** 2021-09-22

**Authors:** Jin-fen Wen, Kai Zhao, Jun-heng Lv, Jin-long Huo, Zi-ran Wang, Hong-jian Wan, Hai-shan Zhu, Zhu-qing Zhang, Gui-fang Shao, Jiao Wang, Shui Zhang, Ting-yu Yang, Jing-rou Zhang, Xue-xiao Zou, Ming-hua Deng

**Affiliations:** 1 College of Horticulture, Yunnan Agricultural University, Kunming, China.; 2 Faculty of Architecture and City Planning, Kunming University of Science and Technology, Kunming, China.; 3 Hunan Academy of Agricultural Science, Changsha, China.; 4 College of Agriculture and Life Sciences, Cornell University, Ithaca, NY, USA.; 5 Faculty of Animal Science and Technology, Yunnan Agricultural University, Kunming, China.; 6 Zhejiang Academy of Agricultural Science, Hangzhou, China.

**Keywords:** *Capsicum annuum* L., comparative transcriptomic analyses, transgenic plants, mitochondrial targeting, VIGS

## Abstract

Cytoplasmic male sterility (CMS) is a maternally inherited trait that derives from the inability to produce functional pollen in higher plants. CMS results from recombination of the mitochondrial genome. However, understanding of the molecular mechanism of CMS in pepper is limited. In this study, comparative transcriptomic analyses were performed using a near-isogenic CMS line 14A (CMS-14A) and a maintainer line 14B (ML-14B) as experimental materials. A total of 17,349 differentially expressed genes were detected between CMS-14A and ML-14B at the PMC meiosis stage. Among them, six unigenes associated with CMS and 108 unigenes involved in energy metabolism were identified. The gene *orf165* was found in CMS-14A. When **orf165** was introduced into ML-14B, almost 30% of transgenic plants were CMS. In addition, *orf165* expression in transgenic CMS plants resulted in abnormal function of some genes involved in energy metabolism. When *orf165* in transgenic CMS plant was silenced, the resulted *orf165*-silenced plant was male fertile and the expression patterns of some genes associated with energy metabolism were similar to ML-14B. Thus, we confirmed that *orf165* influenced CMS in pepper.

## Introduction

Pepper (*Capsicum annuum* L.) is originally from Central and South America. It is one of the most important vegetables in the world (Zou, 2002). Pepper has very obvious heterosis. At present, commercial hybrid pepper seeds are mainly produced by manual emasculation and artificial pollination (Zou, 2002). Artificial production of hybrid seeds is not only laborious and time-consuming, which increases the cost of hybrid seeds, it also makes it difficult to guarantee the purity of seeds (Dhaliwal and Jindal, 2014). The use of cytoplasmic male sterility (CMS) and insect pollination reduces the labor required, and the cost of hybrid seeds by 50% (Zou, 2002). CMS also improves the purity of F_1_ seeds because self-pollination does not occur. As a result, researchers and breeders are increasingly using CMS and studying its application in hybrid seed production (Dhaliwal and Jindal, 2014).

CMS is a maternal hereditary trait, and CMS plants cannot produce functional pollen grains. Mitochondria are energy-producing organelles, and CMS is generally thought to result from rearrangement of the mitochondrial genome due to the production of a novel open reading frame (ORF) (Hanson and Bentolila, 2004; Linke and Börner, 2005; Kim et al., 2007; Yang et al., 2010). The new mitochondrial ORF consists of fragments derived from other genes, or by deletion of normal mitochondrial genes, such as *urf13* in maize CMS-T (Dewey et al., 1986) and *orf220* in stem mustard (Yang *et al.,* 2010). The relationship between CMS-related ORFs and CMS occurrence has been confirmed in some experiments (Hanson and Bentolila, 2004). In sunflower, transgenic plants expressing the CMS-related *orfH522* gene were male sterile. In addition, transgenic sterile plants showed abnormalities in the tapetum cell layer. In the anther of sterile plants, premature DNA fragmentation and programmed cell death were observed during meiosis (Nizampatnam et al., 2009). In *Brassica juncea*, the mitochondrial targeting sequences of the CMS-associated *orf220* and the β-subunit of the *F*_*1*_ -ATPase induced male sterility. In addition, abnormal flower development was observed in transgenic plants (Yang *et al.,* 2010). In some cases, targeting novel ORFs expressed in mitochondria can lead to male sterility or semi-sterility (Duroc et al., 2006; Kim *et al.,* 2007; Yamamoto et al., 2008; Wang et al., 2016). Most ORFs in the CMS line cause abnormalities energy metabolism in CMS. Molecular studies have shown that changes in the F_1_F_0_-ATP synthase subunit, such as from Honglian rice *ATP6* and sunflower *ATP8* and *ATPA*, may induce CMS in plants (Sabar et al., 2003; Yang *et al.,* 2009a,b; Zheng et al., 2012). For example, *orf79* in rice encodes a cytotoxic peptide co-transcribed with the *B-atp6* gene (Wang *et al.,* 2016), and *orf522* in sunflower encodes a protein homologous to *ATP8* in *Reclinomonas* (Balk and Leaver, 2001). Furthermore, male sterility can be induced by mutation of a gene that encodes energy-metabolizing enzyme subunits. For example, in the *Arabidopsis MGP1* gene, the *F*_*A*_ d subunit mutation of *F*_*1*_ F_*0*_ -ATP synthase causes pollen grains to die (Li *et al.,* 2010).

In pepper, CMS was first seen in *C. annuum* accession PI164835 (Peterson, 1958). Studies have shown that the gene *orf456* is related to CMS of pepper (Kim et al., 2007). Expression of *orf456* in transgenic *Arabidopsis* resulted in male sterility in half of the T1 transgenic population (Kim *et al.,* 2007). The pseudogene Ψatp6-2 is related to *Capsicum* CMS (Kim and Kim, 2006). Male sterility was observed in the maintainer line in which *atp6-2* was silenced (Ji et al., 2014); however, silencing Ψatp6-2 in the CMS line resulted in fertility recovery. The altered transcript *orf507* is reportedly related to CMS in pepper (Gulyas et al., 2010). The *orf507* encodes a toxic protein that interacts with a subunit of ATP synthase 6 to inhibit cell growth (Li et al., 2013). The expression of *orf507* in the pepper maintainer line resulted male sterility in transgenic plants (Ji *et al.,* 2015).

Using next-generation sequencing technology (RNA-Seq), whole transcriptome sequencing is a convenient way to study gene expression at the genome-wide level and to define presumed gene functions. In recent years, many studies have demonstrated the efficiency and sensitivity of RNA-Seq in variety of biological contexts (Liu et al., 2013; Martínez-López et al., 2014). Rapid progress has been made in understanding transcriptional procedures related to the specific developmental processes of many plant species, but little study has been conducted in pepper (Liu *et al.,* 2013; Martínez-López *et al.,* 2014). There is a great opportunity to do this kind of research using RNA-Seq in pepper.

CMS line 14A (CMS-14A) was generated by backcrossing several generations of 14B natural CMS mutants. Microscopic examination showed that the anthers in CMS-14A were abnormally developed during meiosis of pollen mother cells and did not produce fertile pollen. However, little is known about the molecular mechanisms of CMS in CMS-14A. We report here our investigation of the CMS-related gene *orf165* in pepper 14A and demonstrate that when introduced with the mitochondrial targeting sequence it produced a male-sterile phenotype in transformed peppers; and silencing *orf165* in transgenic male-sterile lines resulted in pollen fertility. Based on this evidence, we propose that *orf165* is a potent candidate gene for determining the male sterility phenotype of CMS in pepper.

## Material and Methods

### Plant material

Near-isogenic *C. annuum* CMS-14A (S/rf/rf genotype) (A) and ML-14B (N/rf/rf) (B) were used in this study. These were planted at the Experimental Station of Yunnan Agricultural University in Kunming, China. Flower buds at the developmental stage of the spore cell division stage (stages A1 and B1) and pollen mother cell (PMC) meiosis stage (stages A2 and B2) were selected for RNA-Seq analysis.

### Cytological observation

Cytological observation of flower bud development was performed by optical microscopy reported by Lee et al. (2008).

### RNA extraction and library preparation for transcriptome analysis

Total RNA isolated from A1, B1, A2 and B2 was used to construct a sequence library. Prior to library construction, RNA quality and quantity were verified and treated with DNase I. After purification of the poly-(A) mRNA, adaptor-ligated fragments were generated, and the desired cDNA fragment range (200 ± 25 bp) was excised from the gel. The cDNA fragment was amplified and sequenced with Solexa using the Illumina HiSeq2000 sequencing platform enriched by the Beijing Genomics Institute (Shenzhen, China) (Martínez-López et al., 2014).

### *De novo* assembly, assessment, and annotation

After filtering the sequence data, transcriptome assembly was performed using Trinity short read assembly software. The unigene sequences were blastx-aligned with the protein databases NR, Swiss-Prot, KEGG, and COG (e-value < 0.00001) (Natale et al., 2000), and the best alignment of the proteins was used to determine the sequence orientation of unigenes. For unigenes that were not comparable within the above four libraries, we used ESTScan software to determine the sequence direction (Iseli et al., 1999; Conesa et al., 2005).

### Differential expression gene (DEG) analysis and validation

The KEGG database and related software applications (http://www.genome.jp/kegg/kegg4.html) were used to analyze metabolic pathways (Nakao et al., 1999; Kanehisa et al., 2008). The transcript expression was calculated by the RPKM method using the following formula: RPKM (A) = 10^6^ × C/(N × L/10^3^) (Mortazavi et al., 2008). The DEGs and P-values between the two samples were screened by the method of Audic and Claverie (1997), and the threshold of the P-value in multiple tests was determined by FDR method. We used “FDR ≤ 0.001 and the absolute value of log2Ratio ≥ 1” as a threshold for judging the significance of gene expression differences.

Fourteen DEGs related to energy metabolism and one male sterility-related gene were selected for quantitative real time PCR (qRT-PCR) validation.

### Physiological-biochemical characteristic analysis

The amount of ATP was determined using the method of John (1970) and Wang et al. (2007). The H^+^-ATPase activity was estimated according to Bisswanger (2005). BiFC assays were performed according to Waadt et al. (2008).

### Overexpression of *orf165* in ML-14B

To construct the vector, a mitochondrial transport sequence from yeast (encoding the first 25 amino acids of coxIV) was cloned (Kim et al., 2007). The *coxIV* pre-sequence and *orf165* sequence were then cloned into the pRI101 vector to obtain the 35S-CXORF (pRI101:35S-coxIV-orf165) for plant transformation (Ji et al., 2015). The *A. tumefaciens*-mediated transformation was performed according to Clough and Bent (1998) except for some minor changes as follows: when *Agrobacterium tumefaciens* of OD_600_ was 0.4-0.6, the cells were collected by centrifugation; they were resuspended with 5.0% sucrose + 0.1% silwet-77 + 0.1 mmol·L^−1^ AS as a suspension to an OD_600_ value of 0.5-1.0. After the red fruits were ripe, the seeds were harvested. Antibiotic selection was applied during seed germination and PCR used to identify seedlings grown from resistant seeds. In transgenic pepper plants, 6-8 leaf stage seedlings were used for molecular detection of the *orf165* cassette.

### Southern blotting

Southern blots were performed using the PCR DIG Probe Synthesis Kit and the DIG Luminescence Detection Kit according to the manufacturer’s instructions.

### VIGS *orf165* in transgenic male-sterile plants

The pTRV vector and *A. tumefaciens* strain GV3101 were prepared for VIGS as described by Lacomme et al. (2003). Transgenic plants were grown by cutting propagation (Zhu et al., 2007). There were 50 cut seedlings and four true leaves used for VIGS inoculation assay. At the 6-8 leaf stage, seedlings were used for molecular detection of VIGS at the RNA level. Seedlings whose expression of *orf165* was significantly lower than CK were identified as positive plants of VIGS; otherwise, they were identified as non-silent plants according to Ji et al. (2014).

### Morphological analysis of pollen grains

Pollen abundance and pollen characteristics were scored after flowering. Anthers from 10 randomly selected flowers were collected, observed, and photographed under an optical microscope. Pollen abortion was tested by I_2_-KI staining (Wang et al., 2016).

### qRT-PCR analysis of *orf165* and genes involved in energy metabolism

Total RNA was isolated from flower buds in spore cell division (stage 1), PMC meiosis (stage 2), mononuclear microspores (stage 3) and mature pollen stage (stage 4). First strand cDNA was synthesized and qRT-PCR performed as described in our previous study (Deng et al., 2018). Relative gene expression was calculated using the 2^−ΔΔCt^ method (Livak and Schmittgen, 2001). All primers used are listed in Table S1. All experiments were repeated three times and *ACTIN* was used as a reference gene.

## Results

### Comprehensive analysis of pollen grains and energy metabolism between CMS-14A and ML-14B

**Figure 1 -:**
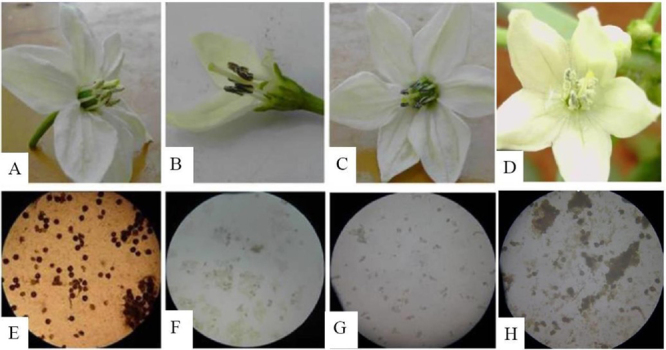
Morphology and pollen viability of TP6, SP23, CMS-14A, and ML-14B. A: Blooming flowers of ML-14B had many pollen gains on the stamen; B: blooming flowers of TP6 had few pollen gains on the stamen; C: blooming flowers of CMS-14A had no pollen gain on the stamen; D: blooming flowers of SP23 had plenty of pollen gains on the stamen; E: most pollen gains from ML-14B appeared round and dark stained with I_2_-KI; F: most pollen gains from TP6 appeared empty and deflated stained with I_2_-KI; G: no pollen gain from CMS-14A was found stained with I_2_-KI; H: most pollen gains from SP23 appeared round and dark stained with I_2_-KI.

ML-14B produced many pollen grains (Figure 1A) but CMS-14A produced none (Figure 1B). I_2_-KI stained the ML-14B pollen grains and they appeared round and dark (i.e. viable) (Figure 1E).

**Figure 2 -:**
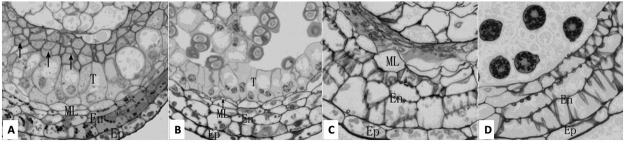
Cytological observation of anthers of cytoplasmic male sterility line (CMS-14A), maintainer line (ML-14B), transgenic male-sterile plant (TP6), and transgenic male-fertility plant (SP-23) (200×). Ep: epidermic cells; En: endothecium cells; T: tapetal cells; ML: middle layer cells. (A). In sterile anthers of CMS-14A (sterility line) at the stage of tetrad microspore, locule did not enlarge and tetrad microspores were extruded and agglutinated (arrows). (B). The fertile anther of ML-14B (maintainer line) at the tetrad stage showed tetrad microspores in locule and anther wall, in which the size of T cells increased and became highly vacuolated. (C). At the time near anthesis, sterile anthers of TP6 (transgenic male-sterile plant) had four empty locules in which only some vestiges of aborted pollen and tapetal cells were apparent. The ML cells were still intact, the wall of En cells showed fiber thickening. (D). In the fertile anther of SP23 (transgenic male-fertility) just before anthesis, both T cells and ML cells degenerated completely and there was one layer of Ep cells and three layers of En cells in the anther wall.

At the meiosis stage of the PMC, the size of tapetal cells of ML-14B increased and they became highly vacuolated (Figure 2B). In CMS-14A, meiosis of the PMC mother cell occurred and the tetrads were formed in the locule, but the tapetal cells were abnormally elongated, bulged, and highly vacuolated; because the locule could not expand, the tetrads were squeezed together (Figure 2A).

**Figure 3 -:**
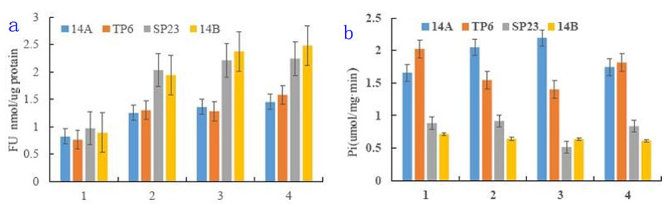
ATP contents and H^+^-ATPase activities in TP6, SP23, CMS-14A, and ML-14B. 1: The spore cell division stage; 2: pollen mother cell meiosis stage; 3: mononuclear microspores; 4: mature pollen stage.

The ATP content in the buds of CMS-14A and ML-14B showed a continuous rising trend, but was significantly lower in CMS-14A than in ML-14B for all stages (Figure 3). The H^+^-ATPase activity was higher in CMS-14A than in ML-14B (Figure 3).

### Flower transcriptome sequence and analysis of DEGs

*Flower transcriptome sequencing output and de novo sequence assembly*

More than 52 × 10^6^ 49-nt clean reads were generated from different development stage of pepper flowers of CMS-14A and ML-14B. Total clean lengths exceeded 47 × 10^6^, and the Q20 alkali content exceeded 97% (base quality value > 20) (Table S2).

Using Trinity, the high quality reads were *de novo* assembled into 140,561, 152,560, 147,640, and 127,553 contigs from A1, A2, B1, and B2, respectively. A total of 97,475 single genes with a total length of 93,202,979 nt were obtained from all libraries.. The size distribution of the pepper flower contigs and unigenes was shown in Table S3.

Using the BLASTX program (E-value < 10^-5^), approximately 60,000 significant BLAST hits were obtained (Figure S1). The ESTScan can be used to predict if a unigene CDS does not have a BLAST hit (Iseli et al., 1999); more than 2000 unigenes were analyzed using this method (Figure S2).

### Functional annotation

BLASTX was used to search distinct gene sequences against the Nr database, and found 61,365 unigenes (63.0% of all 97,475 unigenes) with hits exceeding the E-value cutoff. Similarly, 37,039 unigenes (38.0%) were identified from the SwissProt database. A total of 76,833 unigenes were annotated in one or more of the databases (78.8%), suggesting that they had relatively conserved functions.

Among 61,365 Nr hits, more than 40,000 sequences were classified by COG and distributed among 25 COG categories (Figure S3). Of the 25 COG categories, “General function prediction only” was the largest category, followed by “Replication, recombination and repair,” and “Transcription.” The smallest category were “Nuclear structure” and “Extracellular structures.”

Using BLAST2GO, we assigned more than 3.0 × 10^5^ unigenes and grouped the terms into the three main GO categories and 55 sub-categories (functional groups) (Figure S4). In each of the three main categories (biological processes, cellular components, and molecular functions), the main terms were “Cellular process,” “Metabolic process,” and “Cell.” About half of the genes fell into the “Biological processes” category.

We mapped the annotated sequences to the reference canonical pathways contained in the KEGG database. In all, nearly 35,000 unigenes were assigned to 128 KEGG pathways. The most representative category was “Metabolic pathways”, and the “Biosynthesis of secondary metabolites” and “Plant-pathogen interaction” pathways were also well represented.

### Statistical analysis of DEGs in CMS-14A and ML-14B during flower development

**Figure 4 -:**
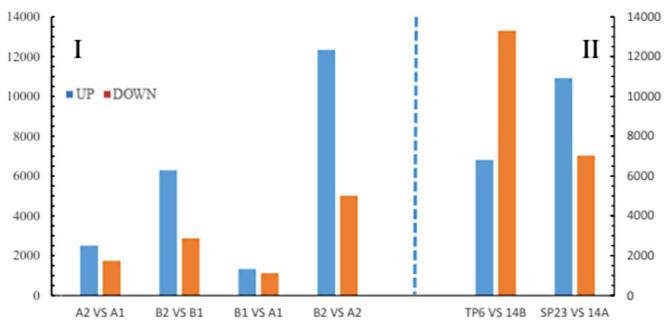
Differentially expressed genes (DEGs).

Based on gene expression profiles, we identified the DEGs between CMS-14A and ML-14B using FDR ≤ 0.001 and the absolute value of log_2_ ratio ≥ 1 as the threshold. As a result, a large number of DEGs were obtained between the A1-vs.-B1, A2-vs.-A1, B2-vs.-B1, and A2-vs.-B2 (Figure 4I).

Because a CMS may be the result of the interactions between several genes with different biological functions, pathway analysis provides a better functional insight into DEGs and identifies the key genes involved. In B2-vs.-B1, A1-vs.-B1, A2-vs.-A1 and A2-vs.-B2, there were 8,955, 2,448, 4,249, and 17,349 DEGs identified, respectively, and these were assigned to 51 GO categories (Figure S5). Among the genes associated with biological processes, 14.9% were associated with cellular processes, 14.3% with metabolic processes, 11.2% with single-organism processes, and 7.6% with response to stimuli in A2-vs.-B2. Among the cellular component, genes encoding cell and cell part were dominant (24.6%), followed by organelles (19.3%) and membranes (10.2%). Catalytic activity (44.1%) and binding (42.2%) were the main functional groups.

### Analysis of DEGs associated with CMS and energy metabolism in pepper flower and qRT-PCR validation

There were 108 unigenes identified as involved in energy metabolism (Table S4). In A2-vs.-B2, B2-vs.-B1, A2-vs.-A1, and A1-vs.-B1, there were 30, 57, 48, and 48 unigenes up-regulated and 15, 12, 19, and 24 down-regulated, respectively (Table S5, S6, S7, S8).

**Figure 5 -:**
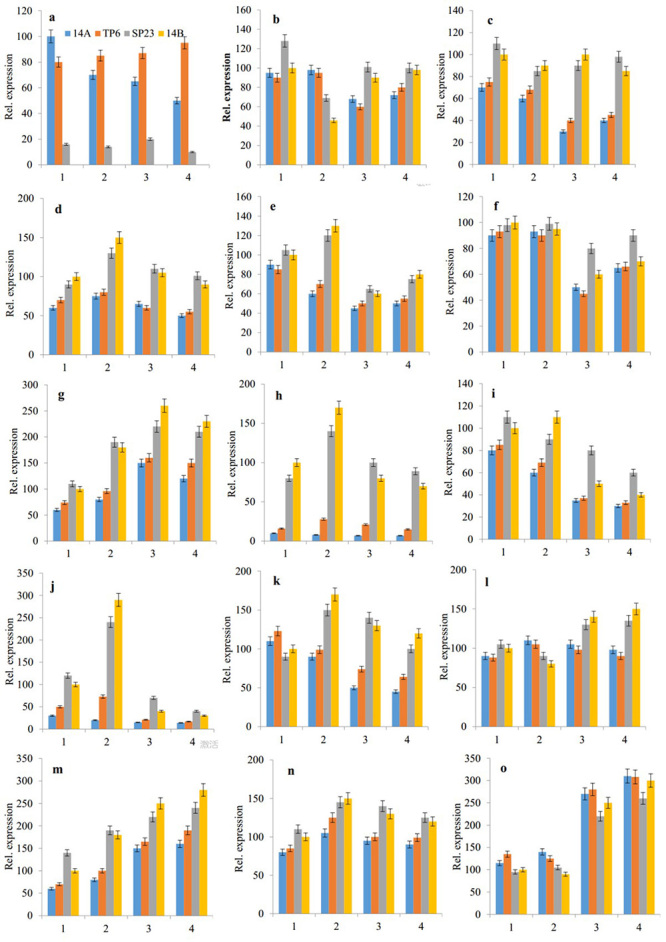
The qRT-PCR analysis of selected CMS-related gene and energy metabolism related genes in CMS-14A, TP6, SP23, and ML-14B. 1: the spore cell division stage; 2: pollen mother cell meiosis stage; 3: mononuclear microspores; 4: mature pollen stage. A: Unigene18482_All; B: Unigene13850_All; C: Unigene11869_All; D: CL5535.Contig2_All; E: CL9996.Contig2_All; F: Unigene2101_All; G: CL420.Contig2_All; H: CL5464.Contig1_All; I: Unigene28657_All; J: CL11399.Contig2_All; K: CL11665.Contig4_All; L: Unigene24327_All; M: CL367.Contig2_All; N: Unigene33404_All; O: CL6833.Contig3_All.

Fourteen unigenes involved in energy metabolism and one unigene associated with CMS were selected for qRT-PCR analysis. The 14 unigenes were putatively the *ATP synthase beta subunit* (*ATP2*), *ATP synthase subunit delta* (*ATP4*), *ATP synthase subunit 6* (*ATP6*), *ATP synthase subunit alpha* (*ATPA*), *cytochrome oxidase subunit 2* (*coxII*), *cytochrome oxidase subunit III* (*coxIII*), mitochondrial *nad2*, mitochondrial *nad7*, apocytochrome *b* (*cob*), *aconitate hydratase*, *succinate dehydrogenase*, and *pyruvate kinase*. The expression patterns of the selected unigenes were basically consistent with Illumina sequencing (Figure 5).

### Characterization of ORF165 from CMS-14A

Six unigenes associated with CMS were identified (Table S4). Compared with ML-14B, CL10138.Contig1, CL1641.Contig1, and CL1641.Contig4 in CMS-14A were down-regulated, but CL10138.Contig2 was up-regulated. Unigene22461 was not expressed in CMS-14A, but was highly expressed in ML-14B. Unigene18482, with known protein function of CMS, was uniquely expressed in CMS-14A, indicating that it may be related to CMS in pepper, so it was selected for further study.

BLAST search showed that the nucleotide and protein sequences of this fragment (Unigene18482) showed high similarity (99% and 98%) to *orf507* (Accession No. FJ175153 and ACJ74044.1) in CMS pepper. According to the previous results of *orf507*, total DNA extracted from CMS-14A and ML-14B was used for PCR. The expected size (about 600 bp) of a single PCR product was obtained in CMS-14A, but no bands were detected in ML-14B. After cloning and sequencing the PCR fragment, a 498-bp ORF encoding 165 amino acids was identified and named *orf165*. Compared with *orf507*, nine nucleotide deletions were detected at the +22 to +30 bp position, and three point-mutations were detected at +34, +39, and +42 bp positions in *orf165*: +39 and +42 bp were silent; and at +34 bp, lysine (AAA) became glutamine (CAA) (Figure S6). It was predicted that the deletion of nine nucleotides would shorten the *orf507* product to 165 amino acids. Multiple alignments showed that *orf165* and *orf507* shared 99% nucleotide sequence identity.

The total base composition of *orf165* CDS was A 31.12% (155), G 19.88% (99), T 32.13% (160), and C 16.87% (84). The theoretical pI and the molecular mass of orf165 were 5.147 and 1.9109 kDa, respectively. The total number of negatively charged residues (Asp + Glu) in orf165 was 19. Total number of positively charged residues (Arg + Lys) in orf165 was 15. The protein contained 876 carbon, 1325 hydrogen, 215 nitrogen, 249 oxygen, and 8 sulfur, with formula C_876_H_1325_N_215_O_249_S_8_. The total number of atoms was 2673, extinction coefficient was 9065 (units of M^−1^ cm^−1^, at 280 nm measured in water), estimated half-life was 30 h (mammalian reticulocytes, *in vitro*), N-terminal of the sequence considered was M (Met), the calculated instability index (II) was 38.75, and aliphatic index was 82.18. The orf165 protein sequence was submitted to SignalP, and had no N-terminal signal peptide and was a non-secretory protein. A transmembrane region was predicted in orf165 (37-59AA). The secondary structure predicted by SOPMA indicated that the deduced orf165 contained 69 alpha helices (41.67%), 51 extended strands (30.95%), 14 beta turns (8.93%), and 31 random coils (18.45%).

The cDNA from the buds in various developmental stages of CMS-14A and ML-14B was used as templates for qRT-PCR analysis to check *orf165* expression. The *orf165* was strongly expressed in CMS-14A, but not in ML-14B (Figure 5). The *orf165* was highly expressed in flowers, but not in seeds, placentas, pericarps, roots, stems, and leaves.

**Figure 6 -:**
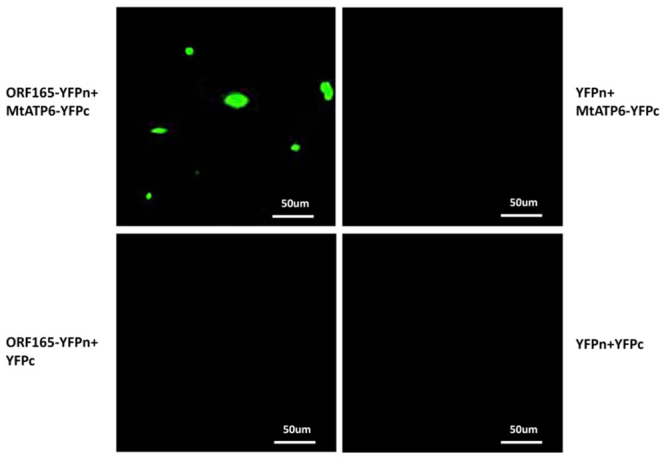
Bimolecular fluorescence complementation (BiFC) assay for *in vivo* interaction of orf165 with MtATP6. YFP fluorescence was observed when ORF165:YFPn was co-expressed with MtATP6:YFPc. No YFP fluorescence was detected in control infiltration of ORF165:YFPn with YFPc, YFPn with MtATP6:YFPc, and YFPn with YFPc.

It was reported that orf507 specifically interacted with MtATP6 (Li et al., 2013). The protein sequence of orf165 shared high similarity (98%) with orf507 and so orf165 may also interact with MtATP6. The PCR was performed using total cDNA extracted from CMS-14A and ML-14B based on the *MtATP6* sequence reported by Li *et al.* (2013). A single PCR product was obtained in CMS-14A and ML-14B. After cloning and sequencing the PCR fragment, a 168-bp ORF encoding 55 amino acids was identified. When plants were transformed with *Agrobacterium* harboring *MtATP6* and *orf165* fused to split halves of the yellow fluorescent protein, yellow fluorescence signals were observed in leaf tissues through confocal microscopy, indicating physical interaction between MtATP6 and orf165 proteins (Figure 6).

### Overexpressed *orf165* in pepper leads to pollen abortion

To determine the correlation between *orf165* and the CMS characters in pepper, two constructs for ML-14B were performed. In the first construct (*coxIV-orf165*), the *orf165* sequence was fused to the yeast’s nuclear *coxIV* pre-sequence (54 codons) for mitochondrial targeting of the protein. The second construct (*coxIV-*non*orf165*) did not contain *orf165*.

The first (*coxIV-orf165*) and second (*coxIV-*non*orf165*) constructs driven by 35S gene promotor were used to transform ML-14B. The floral phenotype of the second (without *orf165*) transformants was normal and similar to that of controls (ML-14B). However, the anthers of *coxIV*-*orf165* plants exhibited a male-sterile phenotype. The pistils of anthers of *coxIV-orf165* transformants developed normally; however, the pistils produced no pollen grains at the dehiscent stage.

About 30% of the total T_1_ transgenic plants (TPs) that contained *coxIV-orf165* showed the male-sterile phenotype. Using the *coxIV-orf165* plasmid as the positive control and the non-transgenic plant (ML-14B) as the negative control, PCR analysis with 17 kanamycin-resistant TPs was examined using gene-specific PCR primers. All TPs could amplify the objective fragment, but no PCR products were observed in non-transgenic plants (Figure S7A). The qRT-PCR analysis of these kanamycin-resistant TPs showed that *orf165* was strongly expressed in male-sterile plants (TP1, 3, 4, 6, 8, 12, 16, and 17); TP2 was kanamycin resistant but slightly expressed the targeted *orf165* and had a semi-male-sterile phenotype; several TPs (TP5, 7, 9, 10, 11, 13, 14, and 15) were kanamycin resistant but had a trace amount of targeted *orf165* expression and a fertile phenotype indistinguishable from controls (Figure S7B).

TP6 was selected for analysis of pollen grains and gene expression. It produced few pollen grains (Figure 1C), while ML-14B produced many (Figure 1A) and CMS-14A produced none (Figure 1B). The pollen grains were stained with I_2_-KI. Compared with the round and dark (i.e. viable) pollen grains of ML-14B, most pollen grains (77.7%) from TP6 appeared empty and deflated (Figure 1E, G). This indicated that TP6 plant was semi-sterile, unlike CMS-14A with no pollen grains (Figure 1F).

**Figure 7 -:**
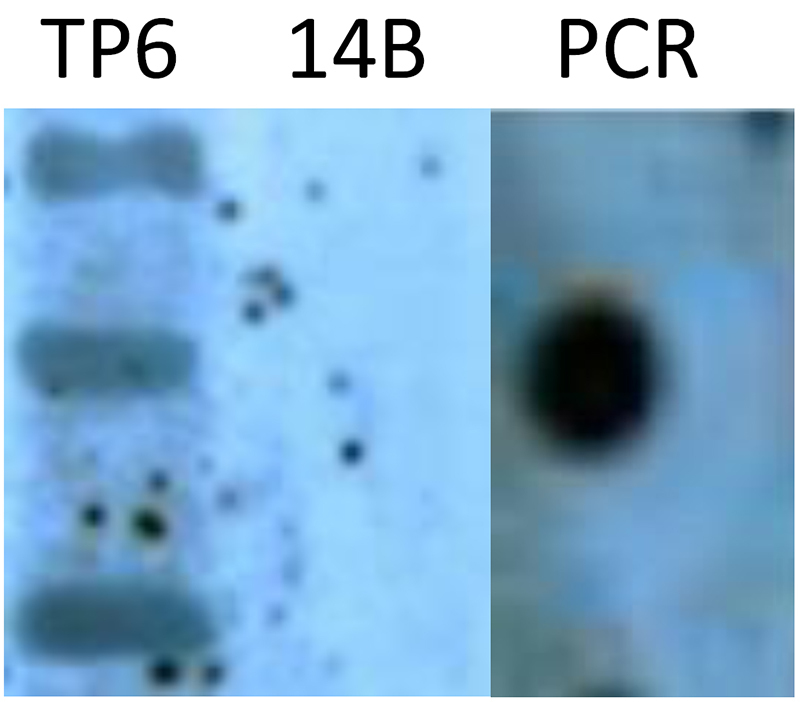
Southern analysis of gDNA digested with *Eco*RI and *Hind*III hybridized with *orf165* probe to check copy number of exogenous gene in transgenic male-sterile plant (TP6). Of total gDNA, 10 μg was digested with *Eco*RI and *Hind*III, separated in a 0.8% agarose gel, and transferred to a Hybond N+ membrane. The probe was digoxygenin (DIG-11-dUTP)-labeled 400-bp orf165. Southern blotting was performed using the PCR DIG Probe Synthesis Kit and DIG Luminescent Detection Kit according to the manufacturer’s instructions. The *orf165* showed three copies in TP6. ML-14B (a maintainer line of CMS 14A) was used as negative control and no band occurred in it; the PCR product of orf165 was used as a positive control.

The qRT-PCR analysis showed that *orf165* expression increased slightly in TP6, decreased gradually in CMS-14A, and was absent from ML-14B throughout all stages of flower development (Figure 5). Southern blotting results showed three copies of *orf165* in TP6 (Figure 7). The microscope image showed that large numbers of PMCs died inside the anthers of TP6 (Figure 2C).

### VIGS of *orf165* restores pollen fertility

We silenced *orf165* in TP6 seedlings using VIGS and then analyzed the pollen abundance, pollen characteristics, and gene expression of VIGS plants.

The flower phenotype of TP6 plants inoculated with pTRV empty vector was abnormal and similar to TP6. However, the anthers of VIGS plants exhibited a male fertile phenotype (Figure 1D).

Fifty plants of TP6 inoculated with VIGS were selected for qRT-PCR assay. Of the 50 VIGS plants, 56% were identified as *orf165*-silenced positive plants (SP). The SP23 (no. 23 *orf165* SP) slightly expressed the targeted *orf165* and had a large number of pollen grains, with a male fertile phenotype indistinguishable from TP6.

SP23 was selected for analysis of pollen grains and gene expression. The results showed that SP23 had a large number of pollen grains (Figure 1D). Pollen grains of SP23 were stained with I_2_-KI (Figure 1H). Most pollen grains of SP23 looked round and dark (i.e. viable), similar to those of ML-14B (Figure 1E).

Both tapetal cells (T) and middle layer cells (ML) degenerated completely and mature pollen grains formed in fertile anthers of SP23 just before anthesis (Figure 2D).

### Manipulating expression of *orf165* affects energy metabolism of flower buds

Using CMS-14A and ML-14B as controls, we analyzed ATP content and H^+^-ATPase activity in flowers of TP6 and SP23. The ATP contents in flowers of TP6 and CMS-14A showed similar trends and were significantly lower than those in ML-14B (Figure 3). The H^+^-ATPase activity in flowers of TP6 and CMS-14A was higher than that in ML-14B (Figure 3). The ATP levels in SP23 were similar to those in ML-14B, but much higher than in CMS-14A and TP6 (Figure 3). The H^+^-ATPase activity in flowers of SP23 was lower than in TP6 and CMS-14A and was similar to that in ML-14B (Figure 3).

The RNA-Seq analyses of TP6 and SP23 were integrated with transcriptomic profiling of CMS-14A and ML-14B at the PMC meiosis stage. The results showed that 6806 and 13,300 DEGs in the TP6 were up- or down-regulated, respectively, compared with the control (ML-14B). Compared with TP6, a total of 10,912 and 7,022 DEGs in SP23 were up- or down-regulated, respectively. Compared with ML-14B, 5,009 and 12,340 DEGs in CMS-14A were up- or down-regulated, respectively. These results indicated that expression of *orf165* had a significant impact on global gene expression. The GO enrichment analysis classified three main categories: “Biological process”, “Molecular function,” and “Cellular component”. In the “Molecular function” category, most DEGs were related to the “Catalytic activity” subcategory, while other DEGs were related to “Electron carrier activity” and “Enzyme regulator activity”. The analyses indicated that *orf165* might be related to energy metabolism.

We analyzed the expression of these genes in TP6 flowers using CMS-14A and ML-14B as controls (Figure 5). In TP6, throughout all stages of flower development, expression of CL6833.Contig3 (*pyruvate kinase*) was up-regulated gradually; while expressions of Unigene11869 (*ATP4*), CL5535.Contig2 (*ATP6*), CL9996.Contig2 (*ATPA*), Unigene2101 (*COXII*), CL420.Contig2 (*COXIII*), CL5464.Contig1 (*NAD2*), Unigene28657 (*NAD7*), CL11399.Contig2 (*COB*), CL11665.Contig4 (*aconitase*), Unigene24327 (*succinate dehydrogenase*), CL367.Contig2 (*succinate dehydrogenase*), and Unigene33404 (*pyruvate kinase*) decreased. Expression of Unigene13850 (*ATP2*) was up-regulated in the second stage and down-regulated in the first, third, and fourth stages. The expressions levels of 14 genes in TP6 were more similar to those in CMS-14A than those of ML-14B. The altered function of these 14 genes might be a factor that induced CMS in pepper.

The qRT-PCR was used to determine the expression of the 14 selected genes involved in energy metabolism in SP23 flowers (Figure 5). Unigene11869, CL5535.Contig2, CL420.Contig2, CL5464.Contig1, and CL11399.Contig2 expression in TP6 was down-regulated, but increased in SP23 and reached similar expression levels in ML-14B. Compared to TP6, the expression of CL6833.Contig3 (pyruvate kinase) in SP23 was down-regulated gradually. The expression levels of 14 genes in SP23 were more similar to those of ML-14B than TP6. These results indicated that *orf165* expression was one factor causing pollen abortion in TP6 and CMS-14A.

## Discussion

### Near-isogenic lines combined with transcriptome sequencing is an effective method to screen for male sterility related genes

Near-isogenic lines combined with transcriptome sequencing can maximally eliminate genetic background variations, reduce false positives, and identify genes that control specificity more accurately and effectively (Liu et al., 2013; Chen et al., 2014). Near-isogenic lines have been widely used in the analysis of CMS in various plants in combination with transcriptome sequencing, and some CMS-related genes have been found (Zheng et al., 2012; Liu *et al.,* 2013; Chen *et al.,* 2014; Yang et al., 2014; Wang et al., 2016). The material CMS-14A used in this study was produced by backcrossing several generations of natural CMS mutants of ML-14B (Zou, 2002). In this case, CMS-14A and ML-14B were a pair of typical near-isogenic lines with the exception of fertility. Transcriptome analysis of the buds indicated that the obtained DEGs were most likely related to CMS. In this study, we obtained a number of DEGs, including a total of 17,349 at the beginning of abortion (A2 vs B2 comparison).

We identified a total of six unigenes associated with CMS by functional annotation of DEGs. Among these genes, unigene 18482_All was shown to encode CMS-related protein by functional annotation, and was only expressed in CMS-14A. Further studies showed that unigene 18482_All only existed in CMS-14A, indicating that it was a new gene. Many studies have shown that amplification, rearrangement, and recombination of mitochondrial genes in higher plants leads to formation of new ORFs and CMS. The first CMS-related gene identified in higher plants was *urf13* in the mitochondrial genome of maize; specific genes associated with CMS were subsequently identified in the mitochondrial genomes of rice, sunflower, *Brassica*, radish, and pepper (Dewey et al., 1986; Singh and Brown, 1993; Dieterich et al., 2003; Kim and Kim, 2006; Wang et al., 2006; Kim *et al.,* 2007; Ashutosh et al., 2008; Lee et al., 2009; Nizampatnam et al., 2009; Das et al., 2010; Jing et al., 2011).

Sequence alignment revealed that the nucleotide sequence of Unigene18482_All was highly similar (99%) to the CMS-related gene *orf507* in mitochondria of CMS-417A in GenBank (Kim et al., 2007; Gulyas et al., 2010). Unigene18482_All had a nine-base deletion (+22 to +30) and a three-base mutation in Unigene18482_All (+34, +39, and +42). It is presumed to encode a 165-amino-acid peptide chain, and was named *orf165*. Male sterility lines from different origins had similar sequences, which have also been reported in *Brassica*; the genes *orf288*, *orf286*, and *orf263* associated with male sterility in *Brassica* had high homology (Jing et al., 2011).

### Overexpression of *orf165* induced male sterility of TPs

To assess the relationship between *orf165* and CMS, *orf165* was introduced into ML-14B. Because *orf165* is thought to be a mitochondrial gene, the pre-sequence of *coxIV* in yeast was used as a signal peptide for *orf165* (Kim et al., 2007; Nizampatnam et al., 2009). The results showed that TPs with the *coxIV* pre-sequence transferred only showed normal fertility, but about 30% of TPs with *coxIV*-*orf165* transferred showed CMS. The anthers of TP6, a typical transgenic male-sterile plant, appeared dry and empty and did not normally break open to distribute pollen grains. The microscopic view of I_2_-KI-stained anthers showed that most TP6 pollen grains were empty, indicating that *orf165* caused CMS in transgenic pepper TP6. Introduction of *orf220* of *Brassica* into plants could result in CMS (Yang et al., 2010); and the introduction of *orf507*, a CMS-related gene of pepper lines, into *Arabidopsis* resulted in about 45% of transgenic *Arabidopsis* plants showing CMS (Kim *et al.,* 2007). Our results are basically consistent with previous reports.

### Silencing of *orf165* restores fertility of transgenic male plants

To verify that CMS of TP6 was caused by *orf165* expression, we silenced *orf165* in TP6 by VIGS technology. SP23, a typical silenced plant, produced a large number of pollen grains, and I_2_-KI staining showed that pollen produced by SP23 was round and black, which was highly similar to ML-14B, indicating that *orf165* silencing could lead to restoration fertility. Previously, according to reports, plants with silencing of the CMS-related gene Ψatp6-2 could form a lot of pollen, but the control plants did not form pollen (Ji et al., 2015). Our results are in line with previous reports.

### Expression of male sterility genes leads to abnormal energy metabolism in anthers

The mitochondrial CMS-related genes interact with certain energy metabolism genes to reduce or inactivate enzyme activity and disrupt energy supply balance, resulting in CMS (Sabar et al., 2003). The CMS-related gene *orf507* of pepper caused microspore abortion by reducing the activity of ATP synthase (Li et al., 2013); Ψatp6-2 can lead to a decrease in ATP synthase, which leads to CMS in pepper (Ji et al., 2014); in rice, *WA352* inhibits expression of *coxII*, and *orfH79*, both of which resulted in abnormal mitochondrial F_1_F_0_-ATPase activity, leading to CMS (Zhang et al., 2007; Luo et al., 2013). Mutation of *MGP1* in *Arabidopsis* can increase the activity of ATP hydrolase, resulting in mitochondrial destruction and CMS (Li *et al.,* 2010).

Through transcriptome analysis, we obtained a large number of DEGs in CMS-14A and ML-14B, which contained 108 encoding genes related to energy metabolism in mitochondria. Among them, 14 genes were verified by qRT-PCR and most of them were down-regulated in CMS-14A. In *orf165*-overexpressing transgenic TP6, the expression patterns of these 14 genes were similar to that of CMS-14A, but significantly differed from its transgene receptor (ML-14B). However, in SP23 plants whose *orf165* was silenced, the expression patterns of these 14 genes were similar to that of ML-14B, but differed from its receptor (TP6). This may be because both CMS-14A and TP6 both contained a normally expressed *orf165*. The *orf165* may interact with energy metabolism genes, resulting in expression imbalances; whereas in SP23, due to the abnormal expression of *orf165*, expression of the above 14 genes tended to be normal, and were consistent with ML-14B. Previous studies have shown that a pepper CMS-related gene, *orf507*, encodes a toxic protein that interacts with ATP6 and decreases ATP synthase activity, thereby causing CMS (Ji et al., 2014). In this paper, *orf165* was highly homologous to *orf507*. The BiFC results showed that orf165 interacted with MtATP6. The H^+^-ATPase activity in CMS-14A and TP6 was higher than that in ML-14B and SP23. This suggested that the *orf165* expression increased ATP hydrolysis activity, leading to energy supply imbalance and microspore abortion.

## Supplementary material

The following online material is available for this article:

Figure S1 ‒ Size distribution of CDS produced by searching unigene sequences against various protein databases.

Figure S2 ‒ Size distribution of EST obtained from the ESTScan results.

Figure S3 ‒ COG function classification of unigenes.

Figure S4 ‒ GO function classification of unigenes.

Figure S5 ‒ Histogram of GO classifications for differentially expressed genes.

Figure S6 ‒ Multiple alignment of DNA sequences of *orf165* and *orf507*.

Figure S7 ‒ PCR and expression analysis of *orf165* expression in 17 transgenic plants.

Table S1 ‒ Oligonucleotides used in this study.

Table S2 ‒ Output statistics for pepper flowers

Table S3 ‒ Statistics of assembly quality for pepper flowers

Table S4 ‒ DEGs involved in energy metabolism and male sterility.

Table S5 ‒ DEGs involved in energy metabolism in A2 vs B2 comparison.

Table S6 ‒ DEGs involved in energy metabolism in B1 vs B2 comparison.

Table S7 ‒ DEGs involved in energy metabolism in A1 vs A2 comparison.

Table S8 ‒ DEGs involved in energy metabolism in A1 vs B1 comparison.
