# Supplementary material for: Orf165 is associated with cytoplasmic male sterility in pepper
Source: Genet Mol Biol. 2021 Sep 22;44(3):e20210030. doi: 10.1590/1678-4685-GMB-2021-0030 (PMC8459829; doi:10.1590/1678-4685-GMB-2021-0030)
Supplement: Figure S6 ‒ [file 1415-4757-GMB-44-3-e20210030-s6.pdf]

Genetics and Molecular Biology

https://doi.org/10.1590/1678-4685-GMB-2021-0030

Supplementary Material to *"Orf165* is associated with cytoplasmic male sterility in Pepper"

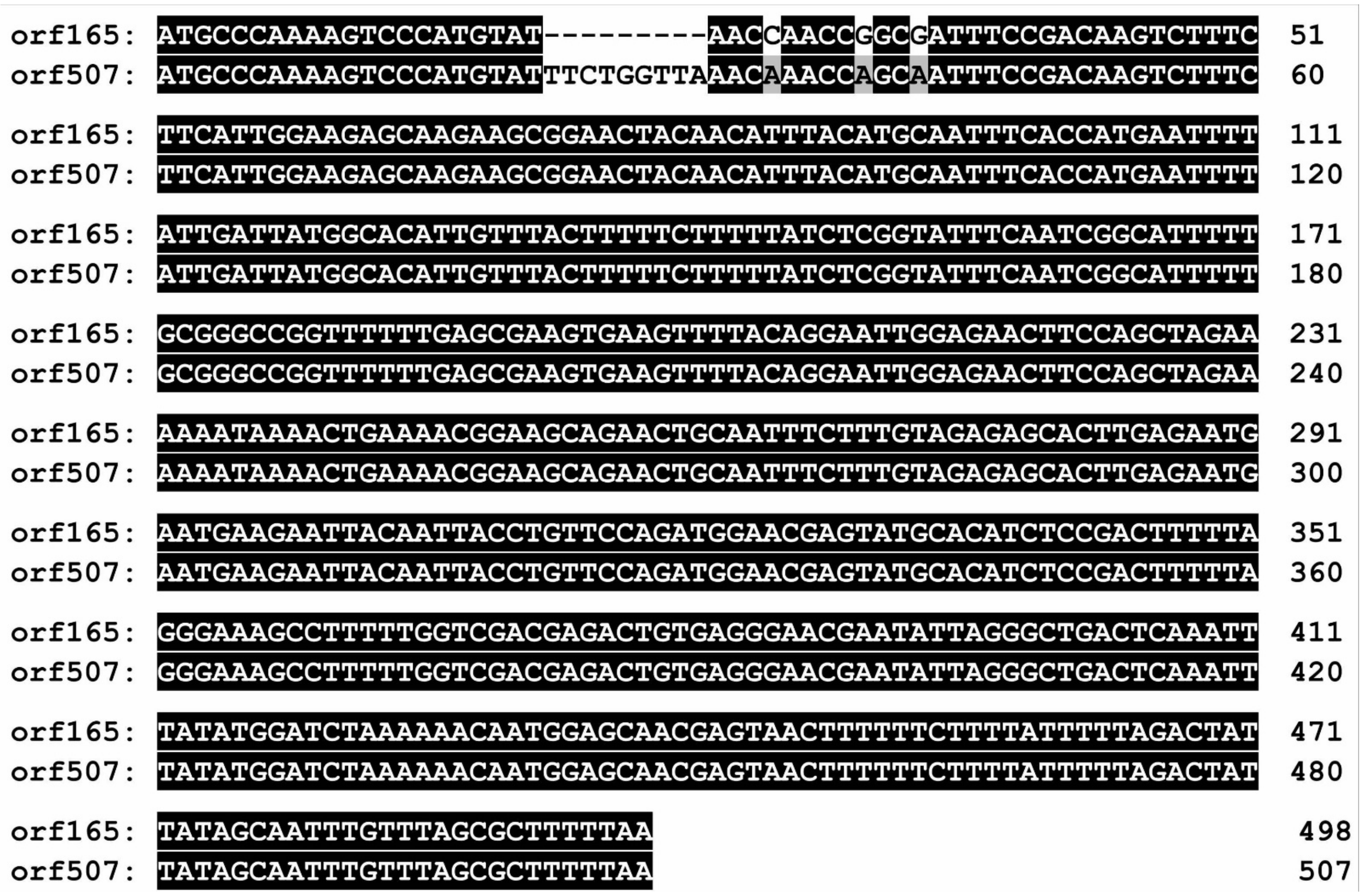

Figure S6 - Multiple alignment of DNA sequences of orf165 and orf507. A nine-nucleotide deletion at the +22 to +30 bp position and three point-mutations at +34, +39, and +42 bp positions were detected in orf165 compared with orf507 (Accession No. FJ175153). The point mutations at +39 and +42 bp were silent; and that at +34 bp changed lysine (AAA) to glutamine (CAA).
