# Supplementary material for: Orf165 is associated with cytoplasmic male sterility in pepper
Source: Genet Mol Biol. 2021 Sep 22;44(3):e20210030. doi: 10.1590/1678-4685-GMB-2021-0030 (PMC8459829; doi:10.1590/1678-4685-GMB-2021-0030)
Supplement: Figure S7 ‒ [file 1415-4757-GMB-44-3-e20210030-s7.pdf]

Genetics and Molecular Biology

https://doi.org/10.1590/1678-4685-GMB-2021-0030

Supplementary Material to *"Orf165* is associated with cytoplasmic male sterility in Pepper"

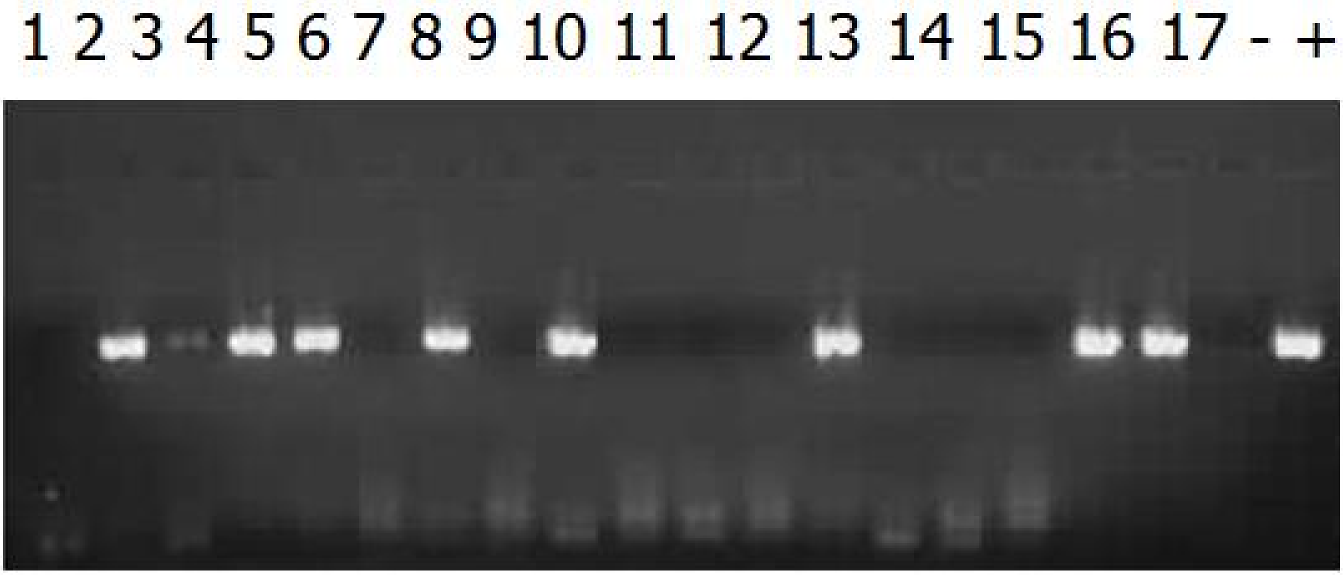

Figure S7 - PCR and expression analysis of orf165 expression in 17 transgenic plants. Seventeen kanamycin-resistant TPs were used for PCR analysis using gene-specific PCR primers with the coxIV–orf165 plasmid as the positive control and the non-transgenic plant (ML-14B) as the negative control. A: All TPs could amplify the objective fragment, but no PCR products were observed in non-transgenic plants. B: Male-sterile plants (TP1, 2, 6, 10, 12, 14, 15, and 17) strongly expressed orf165; TP3, 4, 5, 7, 8, 9, 11, and 13 were kanamycin resistant but had a trace amount of targeted orf165 expression and a fertile phenotype indistinguishable from controls; TP2 was kanamycin resistant but slightly expressed the targeted orf165. C: The ACTIN expression was the internal control.
