# Supplementary material for: Orf165 is associated with cytoplasmic male sterility in pepper
Source: Genet Mol Biol. 2021 Sep 22;44(3):e20210030. doi: 10.1590/1678-4685-GMB-2021-0030 (PMC8459829; doi:10.1590/1678-4685-GMB-2021-0030)
Supplement: Table S1 ‒ [file 1415-4757-GMB-44-3-e20210030-s8.pdf]

Genetics and Molecular Biology
https://doi.org/10.1590/1678-4685-GMB-2021-0030

Supplementary Material to "*Orf165* is associated with cytoplasmic male sterility in Pepper"

Table S1 - Oligonucleotides used in this study. The restriction enzyme sequences for cloning are indicated by underline and bold.

Primer names	Forward (5′–3′)	Reverse (5′–3′)	Application
Unigene11869	AAGGGCCTCACAGAGTTCACA	AAGGGCCTCACAGAGTTCACA	qRT-PCR
CL367.Contig2	GAGTGCAGCAGGACCAAGATAA	ACCGCAGAGTAAGAGTGATAGGG	qRT-PCR
CL420.Contig2	ACCCGTGGAGGTAGAAAGGTTG	TACTGGCTGGCTAGGCTGCTGT	qRT-PCR
CL6833.Contig3	TAAACAAATAAACCAAAAGTATTCC	GGAATACTTTTGGTTTATTTGTTTA	qRT-PCR
CL11665.Contig4	GGGTACTTTCGCCAATATCCGTCTT	CCAATCTCGGGAGCTTCCACTA	qRT-PCR
CL11399.Contig2	GCACGAGTCAGAAACCGATGTA	GACCTGCTGCGGAGCTAACCCT	qRT-PCR
CL5464.Contig1	AAAGCCTCCTACCCTAATACTATGC	CAACCGTTTGACTTTGGCACAT	qRT-PCR
Unigene13850	GCTTTTCCTGTATGTTTCTGGC	TCAAACCTGTAAGTTCAGCACG	qRT-PCR
Unigene33404	GACATCCTGCTGAATCTACCAA	AGCCTCTAACAACACCCTAATC	qRT-PCR
Unigene24327	TTCTCGGCAACATCTGCTCATA	TTTCCATAAATCGCTCACCCTC	qRT-PCR
Unigene28657	CTGTGCCGAGATGTGAGGTGC	GAAGGGAGAAGGTTGTGAAGGTG	qRT-PCR
Unigene2101	GAACGGTGGGAGGTCACAGAG	AGCGTGCATTATGCCAACAGG	qRT-PCR
CL9996.Contig2	CCGTTATTGCTGCTGGATTGG	TTCCCTCTGCCTCAGGCTGTC	qRT-PCR
CL5535.Contig2	ATTTCGTATAACGGAACTCAG	ATTTCCAGAAAGACCACCTAT	qRT-PCR
actin	TGCAGGAATCCACGAGACTAC	TACCACCACTGAGCACAATGTT	qRT-PCR
orf165	ATGCCCAAAAGTCCCATGT	TTAAAAAGCGCTAAACAAATTG	orf165 CDS
E-orf165	CCG**GAATTC**CAGCCTAGCTCGACCCAA	CCC**AAGCTT**GCCTCCATCCTCCGTTAT	pET32α

coxIV	**GGATCC**ATGTTGTCACTACGTCAATCTATAAGA	**CCCGGG**ACCCTCTTTAGCACCAGGACC	pRI101
p-orf165	**CCCGGG** ATGCCCAAAAGTCCCATGT	**GAGCTC**TTACTCGGTTGCTCCATTGTT	pRI101
VIGS	GAGCAAGAAGCGGAACTAC	TTCGTTCCCTCACAGTCTC	VIGS
