# Supplementary material for: Orf165 is associated with cytoplasmic male sterility in pepper
Source: Genet Mol Biol. 2021 Sep 22;44(3):e20210030. doi: 10.1590/1678-4685-GMB-2021-0030 (PMC8459829; doi:10.1590/1678-4685-GMB-2021-0030)
Supplement: Table S2 ‒ [file 1415-4757-GMB-44-3-e20210030-s9.pdf]

Genetics and Molecular Biology
https://doi.org/10.1590/1678-4685-GMB-2021-0030

Supplementary Material to *"Orf165* is associated with cytoplasmic male sterility in Pepper"

Table S2 - Output statistics for pepper flowers.

Sample	Total raw reads	Total clean reads	Total clean nucleotides (nt)	Q20 percentage
A1	56,006,268	52,626,820	4,736,413,800	97.34
A2	56,528,970	52,841,092	4,755,698,280	97.24
B1	57,826,112	53,911,440	4,852,029,600	97.13
B2	59,057,626	55,408,880	4,986,799,200	97.37
