# Supplementary material for: Orf165 is associated with cytoplasmic male sterility in pepper
Source: Genet Mol Biol. 2021 Sep 22;44(3):e20210030. doi: 10.1590/1678-4685-GMB-2021-0030 (PMC8459829; doi:10.1590/1678-4685-GMB-2021-0030)
Supplement: Table S3 ‒ [file 1415-4757-GMB-44-3-e20210030-s10.pdf]

Genetics and Molecular Biology
https://doi.org/10.1590/1678-4685-GMB-2021-0030

Supplementary Material to "*Orf165* is associated with cytoplasmic male sterility in Pepper"

Table S3 - Statistics of assembly quality for pepper flower.

	Sample	Total Number	Total length (nt)	Mean length (nt)	N50	Total consensus sequences	Distinct clusters	Distinct singletons
	A1	140,561	48,476,780	345	632			
	A2	152,560	51,510,454	338	614			
contig	B1	147,640	50,014,106	339	633			
	B2	127,553	45,583,704	357	673			
	A1	82,215	56,078,296	682	1220			
	A2	88,017	61,989,409	704	1287			
unigenes	B1	82,718	58,197,759	704	1262	82,718	26,428	56,290
	B2	77,061	50,601,412	657	1137	77,061	22,788	54,273
	All	97,475	93,202,979	956	1555	97,475	39,092	58,383
