# Supplementary material for: Orf165 is associated with cytoplasmic male sterility in pepper
Source: Genet Mol Biol. 2021 Sep 22;44(3):e20210030. doi: 10.1590/1678-4685-GMB-2021-0030 (PMC8459829; doi:10.1590/1678-4685-GMB-2021-0030)
Supplement: Table S4 ‒ [file 1415-4757-GMB-44-3-e20210030-s11.pdf]

Genetics and Molecular Biology
https://doi.org/10.1590/1678-4685-GMB-2021-0030

Supplementary Material to *"Orf165* is associated with cytoplasmic male sterility in Pepper"

Table S4 - DEGs involved in energy metabolism and male sterility

geneID	Gene Length	Nr-annotation	Nt-ID	Nt-annotation
atp1				
Unigene17921	455	--	gi|334691741|gb|HQ385215.1|	Hydrophyllum capitatum ATPase alpha subunit (atp1) gene, partial cds; mitochondrial
atp2				
Unigene13850	212	hypothetical protein VITISV_018718 [Vitis vinifera]	gi|19684|emb|X02868.1|	Nicotiana plubaginifolia atp2-1 gene for mitochondrial ATP synthase beta subunit
atp4				
Unigene11535	503	ATP synthase subunit delta', mitochondrial-like [S. lycopersicum]	gi|460390425|ref|XR_182837.1|	PREDICTED: Solanum lycopersicum ATP synthase subunit delta', mitochondrial-like (LOC101245173), misc_RNA
Unigene11869	414	ATP synthase subunit delta', mitochondrial-like [Solanum lycopersicum]	gi|460378903|ref|XM_004235159.1|	PREDICTED: Solanum lycopersicum ATP synthase subunit delta', mitochondrial-like (LOC101248556), mRNA
Unigene25039	422	RecName: Full=ATP synthase subunit delta', mitochondrial;	gi|460390425|ref|XR_182837.1|	PREDICTED: Solanum lycopersicum ATP synthase subunit delta', mitochondrial-like (LOC101245173), misc_RNA
Unigene25237	1084	ATP synthase subunit delta', mitochondrial-like [Solanum lycopersicum]	gi|460388646|ref|XM_004239930.1|	Solanum lycopersicum ATP synthase subunit delta', mitochondrial-like, mRNA
Unigene28876	526	ATP synthase subunit delta', mitochondrial-like [Solanum lycopersicum]	gi|460378903|ref|XM_004235159.1|	PREDICTED: Solanum lycopersicum ATP synthase subunit delta', mitochondrial-like (LOC101248556), mRNA
CL11262.Contig1	948	ATP synthase D chain, mitochondrial, putative	gi|460411738|ref|XM_0	PREDICTED: Solanum lycopersicum ATP synthase subunit d,

geneID	Gene Length	Nr-annotation	Nt-ID	Nt-annotation
		[Solanum demissum]	04251215.1|	mitochondrial-like (LOC101248453), mRNA
atp5				
CL11262.Contig1	948	ATP synthase D chain, mitochondrial, putative [Solanum demissum]	gi|460411738|ref|XM_004251215.1|	PREDICTED: Solanum lycopersicum ATP synthase subunit d, mitochondrial-like (LOC101248453), mRNA
atp7				
Unigene1469	1248	probable ATP synthase 24 kDa subunit, mitochondrial-like [Solanum lycopersicum]	gi|460415838|ref|XM_004253213.1|	PREDICTED: Solanum lycopersicum probable ATP synthase 24 kDa subunit, mitochondrial-like (LOC101262000), mRNA
Unigene30340	474	PREDICTED: oleosin Bn-III-like [Solanum lycopersicum]	gi|460393177|ref|XM_004242140.1|	PREDICTED: Solanum lycopersicum oleosin Bn-III-like (LOC101263037), mRNA
ATP6				
CL5535.Contig1	533	PREDICTED: ATP synthase subunit a-like, partial [Solanum lycopersicum]	gi|913146|gb|S75449.1|	Petunia axillaris subsp. parodii atp6 gene, complete cds; mitochondrial gene for mitochondrial product
CL5535.Contig2	404	PREDICTED: ATP synthase subunit a-like, partial [Solanum lycopersicum]	gi|913146|gb|S75449.1|	Petunia axillaris subsp. atp6 gene, complete cds; mitochondrial gene for mitochondrial product
Unigene13749	367	ATP synthase subunit 6-2 [Capsicum annuum]	gi|71724855|gb|DQ126681.1|	Capsicum annuum ATP synthase subunit 6-2 (atp6-2) gene, complete cds; mitochondrial
Unigene24614	627	PREDICTED: oleosin 5-like [Solanum lycopersicum]	gi|460381016|ref|XM_004236201.1|	PREDICTED: Solanum lycopersicum oleosin 5-like (LOC101263398), mRNA
Unigene32029	817	ATPase subunit 6 [Hibbertia cuneiformis]	gi|71724857|gb|DQ126682.1|	Capsicum annuum ATP synthase subunit 6-1 (atp6-1) gene, complete cds; mitochondrial
Unigene32030	1842	ATP synthase subunit 6-1 [Capsicum annuum]	gi|71724857|gb|DQ126682.1|	Capsicum annuum ATP synthase subunit 6-1 (atp6-1) gene, complete cds; mitochondrial
Unigene32031	1046	ATP synthase subunit 6-1 [Capsicum annuum]	gi|71724857|gb|DQ126682.1|	Capsicum annuum ATP synthase subunit 6-1 (atp6-1) gene, complete cds; mitochondrial

geneID	Gene Length	Nr-annotation	Nt-ID	Nt-annotation
ATP9				
Unigene14245	346	--	gi|37896205|gb|AY305264.1|	Cucumis melo clone atp9 genes, complete sequence; and atp6 pseudogene, partial sequence; mitochondrial
ATPA				
CL6700.Contig3	5885	ATP synthase F1 subunit 1 (mitochondrion) [Nicotiana tabacum]	gi|56806513|dbj|BA000042.1|	Nicotiana tabacum mitochondrial DNA, complete genome
CL9996.Contig1	3665	ATP synthase CF1 alpha subunit (chloroplast) [Capsicum annuum]	gi|401065910|gb|JX270811.1|	Capsicum annuum chloroplast, complete genome
CL9996.Contig2	4420	ATP synthase CF1 alpha subunit (chloroplast) [Capsicum annuum]	gi|401065910|gb|JX270811.1|	Capsicum annuum chloroplast, complete genome
COXII				
CL1985.Contig4	466	--	gi|4106848|gb|AF096321.1|	Solanum tuberosum coxII gene, partial cds; mitochondrial genes encoding mitochondrial proteins
Unigene11642	614	cytochrome oxidase subunit 2 (mitochondrion) [Nicotiana tabacum]	gi|238909246|gb|FJ986190.1|	Capsicum annuum cytochrome oxidase subunit 2 (COXII) gene, partial cds; mitochondrial
Unigene26984	591	cytochrome oxidase subunit 2 (mitochondrion) [Nicotiana tabacum]	gi|238909246|gb|FJ986190.1|	Capsicum annuum cytochrome oxidase subunit 2 (COXII) gene, partial cds; mitochondrial
Unigene2101	901	hypothetical protein VITISV_000627 [Vitis vinifera]	gi|71724859|gb|DQ126683.1|	Capsicum annuum cytochrome oxidase subunit 2 (coxII) gene, complete cds; mitochondrial
Unigene32293	600	coxII (mitochondrion) [Malus x domestica]	gi|13327|emb|X17395.1|	Petunia coxII-2 mitochondrial gene for cytochrome oxidase subunit II
COXIII				
CL420.Contig1	860	--	gi|21325919|gb|AF280607.1|	Solanum tuberosum coxIII gene, complete cds; mitochondrial genes for mitochondrial products
CL420.Contig2	905	--	gi|21325919|gb|AF280	Solanum tuberosum coxIII gene, complete cds; mitochondrial genes for

geneID	Gene Length	Nr-annotation	Nt-ID	Nt-annotation
			607.1|	mitochondrial products
Unigene20200	358	--	gi|21325919|gb|AF280607.1|	Solanum tuberosum coxIII gene, complete cds; mitochondrial genes for mitochondrial products
NAD1				
Unigene10713	345	--	gi|261873505|gb|FJ670426.1|	Eliea articulata NADH dehydrogenase subunit 1 (nad1) gene, exons 2, 3 and partial cds; mitochondrial
Unigene23825	673	unknown [Solanum tuberosum]	gi|158251913|gb|EU049652.1|	Corythophora alta isolate Cal1202_28 NADH dehydrogenase subunit 1 (NAD1) gene, partial cds; mitochondrial
NAD2				
CL1003.Contig14	2225	putative ethylene-responsive element binding protein [Capsicum annuum]	gi|3334857|emb|X93575.1|	Solanum tuberosum mitochondrial trnC, trnN1, trnY, nad2 genes
CL5464.Contig1	2360	hypothetical protein GlmaxMp13 (mitochondrion) [Glycine max]	gi|3334857|emb|X93575.1|	Solanum tuberosum mitochondrial trnC, trnN1, trnY, nad2 genes
CL5464.Contig2	1861	RNA polymerase beta'' subunit (chloroplast) [Capsicum annuum]	gi|3334857|emb|X93575.1|	Solanum tuberosum mitochondrial trnC, trnN1, trnY, nad2 genes
CL8822.Contig1	2464	NADH dehydrogenase subunit [Medicago truncatula]	gi|3334857|emb|X93575.1|	Solanum tuberosum mitochondrial trnC, trnN1, trnY, nad2 genes
CL8822.Contig2	3283	NADH dehydrogenase subunit [Medicago truncatula]	gi|3334857|emb|X93575.1|	Solanum tuberosum mitochondrial trnC, trnN1, trnY, nad2 genes
NAD3				
CL10232.Contig1	599	NADH-ubiquinone oxidoreductase chain [Medicago truncatula]	gi|4106839|gb|AF095279.1|	Solanum tuberosum nad3genes, mitochondrial genes encoding mitochondrial proteins, complete cds
CL8634.Contig1	691	NADH-ubiquinone oxidoreductase chain [Medicago truncatula]	gi|87248034|gb|DQ381456.1|	Beta vulgaris subsp. vulgaris nad3 mRNAs, complete cds; mitochondrial

geneID	Gene Length	Nr-annotation	Nt-ID	Nt-annotation
NAD7				
CL11266.Contig1	1026	unnamed protein product [Vitis vinifera]	gi|56806513|dbj|BA000042.1|	Nicotiana tabacum mitochondrial DNA, complete genome
CL1540.Contig1	1467	conserved hypothetical protein [Ricinus communis]	gi|56806513|dbj|BA000042.1|	Nicotiana tabacum mitochondrial DNA, complete genome
CL1540.Contig2	3066	PREDICTED: NADH dehydrogenase [Solanum lycopersicum]	gi|56806513|dbj|BA000042.1|	Nicotiana tabacum mitochondrial DNA, complete genome
Unigene11959	360	conserved hypothetical protein [Ricinus communis]	gi|56806513|dbj|BA000042.1|	Nicotiana tabacum mitochondrial DNA, complete genome
Unigene28657	909	hypothetical protein NitaMp153 [Nicotiana tabacum]	gi|56806513|dbj|BA000042.1|	Nicotiana tabacum mitochondrial DNA, complete genome
Unigene32075	1986	NADH dehydrogenase subunit 7 (mitochondrion) [Nicotiana tabacum]	gi|56806513|dbj|BA000042.1|	Nicotiana tabacum mitochondrial DNA, complete genome
NAD9				
Unigene14262	348	subunit 9 of NADH dehydrogenase [Arabidopsis thaliana]	gi|56791601|gb|AY832221.1|	Eschscholzia californica NADH dehydrogenase subunit 9 (nad9) gene, partial cds; mitochondrial
Unigene15339	678	NADH dehydrogenase subunit 9 (mitochondrion) [Nicotiana tabacum]	gi|56806513|dbj|BA000042.1|	Nicotiana tabacum mitochondrial DNA, complete genome
COB				
CL11399.Contig1	537	ribosomal protein S14 (mitochondrion) [Nicotiana tabacum]	gi|4106829|gb|AF095274.1|	Solanum tuberosum apocytochrome b (cob) gene, partial cds; mitochondrial genes encoding mitochondrial proteins
CL11399.Contig2	1885	apocytochrome b (mitochondrion) [Millettia pinnata]	gi|4106829|gb|AF095274.1|	Solanum tuberosum r apocytochrome b (cob) gene, partial cds; mitochondrial genes encoding mitochondrial proteins
CL1985.Contig1	818	unnamed protein product [Vitis vinifera]	gi|56806513|dbj|BA000042.1|	Nicotiana tabacum mitochondrial DNA, complete genome

geneID	Gene Length	Nr-annotation	Nt-ID	Nt-annotation
aconitase				
CL11665.Contig1	6561	putative aconitase [Capsicum chinense]	gi|460395805|ref|XM_004243424.1|	PREDICTED: Solanum lycopersicum aconitate hydratase, cytoplasmic-like (LOC101254008), mRNA
CL11665.Contig2	2559	aconitate hydratase 3 [Citrus clementina]	gi|359494042|ref|XM_002278102.2|	PREDICTED: Vitis vinifera aconitate hydratase 2, mitochondrial-like (LOC100253811), mRNA
CL11665.Contig3	3731	putative aconitase [Capsicum chinense]	gi|460395805|ref|XM_004243424.1|	PREDICTED: Solanum lycopersicum aconitate hydratase, cytoplasmic-like (LOC101254008), mRNA
CL11665.Contig4	3570	putative aconitase [Capsicum chinense]	gi|460395805|ref|XM_004243424.1|	PREDICTED: Solanum lycopersicum aconitate hydratase, cytoplasmic-like (LOC101254008), mRNA
CL7127.Contig2	527	RecName: Full=Aconitate hydratase; Short=Aconitase;	gi|156070766|gb|EF517792.1|	Capsicum frutescens BAC 215H17, complete sequence
CL7127.Contig3	875	RecName: Full=Aconitate hydratase; Short=Aconitase;	gi|156070766|gb|EF517792.1|	Capsicum frutescens BAC 215H17, complete sequence
CL7127.Contig4	1085	RecName: Full=Aconitate hydratase; Short=Aconitase;	gi|156070766|gb|EF517792.1|	Capsicum frutescens BAC 215H17, complete sequence
Unigene25633	290	PREDICTED: aconitate hydratase 2, mitochondrial [Vitis vinifera]	gi|359494042|ref|XM_002278102.2|	PREDICTED: Vitis vinifera aconitate hydratase 2, mitochondrial-like (LOC100253811), mRNA
AGPase				
CL2979.Contig1	1135	RecName: Full=Glucose-1-phosphate adenylyltransferase large subunit 2, chloroplastic/amyloplastic;	gi|400488|emb|X74982.1|	S.tuberosum (Desiree) agpS2 mRNA
GAPDH				
CL4065.Contig1	519	glyceraldehyde-3-phosphate dehydrogenase B, chloroplastic-like [Solanum lycopersicum]	gi|327198778|emb|FN691929.1|	S. tuberosum subsp. tuberosum cv. Dessiree mRNA for chloroplast gapdh gene
Succinate				

geneID	Gene Length	Nr-annotation	Nt-ID	Nt-annotation
dehydrogenase				
CL1348.Contig10	2564	PREDICTED: L-aspartate oxidase 1-like [Solanum lycopersicum]	gi|460413044|ref|XM_004251858.1|	PREDICTED: Solanum lycopersicum L-aspartate oxidase 1-like (LOC101264098), mRNA
CL1348.Contig11	2588	PREDICTED: L-aspartate oxidase 1-like [Solanum lycopersicum]	gi|460413044|ref|XM_004251858.1|	PREDICTED: Solanum lycopersicum L-aspartate oxidase 1-like (LOC101264098), mRNA
CL1348.Contig12	2544	PREDICTED: L-aspartate oxidase 1-like [Solanum lycopersicum]	gi|460413044|ref|XM_004251858.1|	PREDICTED: Solanum lycopersicum L-aspartate oxidase 1-like (LOC101264098), mRNA
CL1348.Contig13	1225	PREDICTED: L-aspartate oxidase 1-like [Solanum lycopersicum]	gi|460413044|ref|XM_004251858.1|	PREDICTED: Solanum lycopersicum L-aspartate oxidase 1-like (LOC101264098), mRNA
CL1348.Contig14	2219	PREDICTED: L-aspartate oxidase 1-like [Solanum lycopersicum]	gi|460413044|ref|XM_004251858.1|	PREDICTED: Solanum lycopersicum L-aspartate oxidase 1-like (LOC101264098), mRNA
CL1348.Contig15	2279	PREDICTED: L-aspartate oxidase 1-like [Solanum lycopersicum]	gi|460413044|ref|XM_004251858.1|	PREDICTED: Solanum lycopersicum L-aspartate oxidase 1-like (LOC101264098), mRNA
CL1348.Contig16	1169	PREDICTED: L-aspartate oxidase 1-like [Solanum lycopersicum]	gi|460413044|ref|XM_004251858.1|	PREDICTED: Solanum lycopersicum L-aspartate oxidase 1-like (LOC101264098), mRNA
CL1348.Contig17	2439	PREDICTED: L-aspartate oxidase 1-like [Solanum lycopersicum]	gi|460413044|ref|XM_004251858.1|	PREDICTED: Solanum lycopersicum L-aspartate oxidase 1-like (LOC101264098), mRNA
CL1348.Contig18	1141	PREDICTED: L-aspartate oxidase 1-like [Solanum lycopersicum]	gi|460413044|ref|XM_004251858.1|	PREDICTED: Solanum lycopersicum L-aspartate oxidase 1-like (LOC101264098), mRNA
CL1348.Contig1	2674	PREDICTED: L-aspartate oxidase 1-like [Solanum lycopersicum]	gi|460413044|ref|XM_004251858.1|	PREDICTED: Solanum lycopersicum L-aspartate oxidase 1-like (LOC101264098), mRNA
CL1348.Contig2	1694	PREDICTED: L-aspartate oxidase 1-like [Solanum lycopersicum]	gi|460413044|ref|XM_004251858.1|	PREDICTED: Solanum lycopersicum L-aspartate oxidase 1-like (LOC101264098), mRNA
CL1348.Contig3	2798	PREDICTED: L-aspartate oxidase 1-like [Solanum lycopersicum]	gi|460413044|ref|XM_004251858.1|	PREDICTED: Solanum lycopersicum L-aspartate oxidase 1-like (LOC101264098), mRNA
CL1348.Contig4	2688	PREDICTED: L-aspartate oxidase 1-like [Solanum lycopersicum]	gi|460413044|ref|XM_004251858.1|	PREDICTED: Solanum lycopersicum L-aspartate oxidase 1-like (LOC101264098), mRNA
CL1348.Contig5	2774	PREDICTED: L-aspartate oxidase 1-like [Solanum	gi|460413044|ref|XM_0	PREDICTED: Solanum lycopersicum L-aspartate oxidase 1-like

geneID	Gene Length	Nr-annotation	Nt-ID	Nt-annotation
		lycopersicum]	04251858.1|	(LOC101264098), mRNA
CL1348.Contig6	1876	PREDICTED: L-aspartate oxidase 1-like [Solanum lycopersicum]	gi|460413044|ref|XM_004251858.1|	PREDICTED: Solanum lycopersicum L-aspartate oxidase 1-like (LOC101264098), mRNA
CL1348.Contig7	2036	PREDICTED: L-aspartate oxidase 1-like [Solanum lycopersicum]	gi|460413044|ref|XM_004251858.1|	PREDICTED: Solanum lycopersicum L-aspartate oxidase 1-like (LOC101264098), mRNA
CL1348.Contig8	2422	PREDICTED: L-aspartate oxidase 1-like [Solanum lycopersicum]	gi|460413044|ref|XM_004251858.1|	PREDICTED: Solanum lycopersicum L-aspartate oxidase 1-like (LOC101264098), mRNA
CL1348.Contig9	2478	PREDICTED: L-aspartate oxidase 1-like [Solanum lycopersicum]	gi|460413044|ref|XM_004251858.1|	PREDICTED: Solanum lycopersicum L-aspartate oxidase 1-like (LOC101264098), mRNA
CL182.Contig2	1072	sdh3 gene product (mitochondrion) [Boea hygrometrica]	gi|222137886|gb|FJ597539.1|	Capsicum frutescens clone BAC PEPBAC043M10, complete sequence
CL367.Contig1	443	succinate dehydrogenase, mitochondrial-like [Solanum lycopersicum]	gi|460372151|ref|XM_004231848.1|	Solanum lycopersicum succinate dehydrogenase, mitochondrial-like (LOC101253337), mRNA
CL367.Contig2	1338	mitochondrial succinate dehydrogenase iron sulfur subunit [Solanum lycopersicum]	gi|225313241|dbj|AK320385.1|	Solanum lycopersicum cDNA, clone: LEFL1008CE05, HTC in leaf
CL420.Contig1	860	--	gi|21325919|gb|AF280607.1|	Solanum tuberosum succinate dehydrogenase, complete sequence; mitochondrial genes for mitochondrial products
CL420.Contig2	905	--	gi|21325919|gb|AF280607.1|	Solanum tuberosum succinate dehydrogenase, complete sequence; mitochondrial genes for mitochondrial products
CL6700.Contig1	2460	succinate dehydrogenase subunit 3 [Solanum lycopersicum]	gi|56806513|dbj|BA000042.1|	Nicotiana tabacum mitochondrial DNA, complete genome
CL6700.Contig4	4142	hypothetical protein BevumaM_p022 [Beta vulgaris subsp. maritima]	gi|56806513|dbj|BA000042.1|	Nicotiana tabacum mitochondrial DNA, complete genome
Unigene12355	966	PREDICTED: uncharacterized protein [Solanum lycopersicum]	gi|460380216|ref|XM_004235806.1|	PREDICTED: Solanum lycopersicum uncharacterized LOC101244854, transcript variant 1 (LOC101244854), mRNA
Unigene12480	828	PREDICTED: succinate dehydrogenase g, mitochondrial-like [Solanum lycopersicum]	gi|460402441|ref|XM_004246673.1|	Solanum lycopersicum succinate dehydrogenase assembly factor 1 homolog, mitochondrial-like (LOC101265269), mRNA
Unigene13723	313	unknown [Glycine max]	gi|76446083|gb|DQ158	Nicotiana clevelandii trypsin proteinase inhibitor precursor (PI) gene,

geneID	Gene Length	Nr-annotation	Nt-ID	Nt-annotation
			203.1|	complete cds
Unigene20200	358	--	gi|21325919|gb|AF280607.1|	Solanum tuberosum succinate dehydrogenase, complete sequence; mitochondrial genes for mitochondrial products
Unigene24327	2604	PREDICTED: succinate dehydrogenase, mitochondrial-like [Solanum lycopersicum]	gi|460373178|ref|XM_004232352.1|	Solanum lycopersicum succinate dehydrogenase, mitochondrial-like, mRNA
Unigene31173	241	PREDICTED: succinate dehydrogenase, mitochondrial-like [S. lycopersicum]	gi|460383779|ref|XM_004237547.1|	PREDICTED: Solanum lycopersicum succinate dehydrogenase, mitochondrial-like (LOC101252979), mRNA
MDH				
Unigene20397	616	putative mitochondrial NAD-dependent malate dehydrogenase [S. tuberosum]	gi|460413006|ref|XM_004251839.1|	PREDICTED: Solanum lycopersicum malate dehydrogenase 1, mitochondrial-like (LOC101258530), mRNA
Unigene25224	1460	mitochondrial malate dehydrogenase [Solanum lycopersicum]	gi|350536644|ref|NM_001247072.1|	Solanum lycopersicum mitochondrial malate dehydrogenase (mMDH), nuclear gene encoding mitochondrial protein, mRNA
Unigene25419	1754	malate dehydrogenase [Solanum lycopersicum]	gi|350537824|ref|NM_001247127.1|	Solanum lycopersicum malate dehydrogenase (mdh), mRNA
pyruvate kinase				
CL6833.Contig1	1933	PREDICTED: pyruvate kinase, cytosolic isozyme-like [Solanum lycopersicum]	gi|460409163|ref|XM_004249961.1|	PREDICTED: Solanum lycopersicum pyruvate kinase, cytosolic isozyme-like (LOC101256147), mRNA
CL6833.Contig2	352	PREDICTED: pyruvate kinase, cytosolic isozyme-like [Solanum lycopersicum]	gi|460402151|ref|XM_004246530.1|	PREDICTED: Solanum lycopersicum pyruvate kinase, cytosolic isozyme-like, transcript variant 1 (LOC101248036), mRNA
CL6833.Contig3	1495	PREDICTED: pyruvate kinase, cytosolic isozyme-like [Solanum lycopersicum]	gi|460408057|ref|XM_004249417.1|	PREDICTED: Solanum lycopersicum pyruvate kinase, cytosolic isozyme-like (LOC101265599), mRNA
CL6857.Contig4	3543	PREDICTED: plastidial pyruvate kinase 4, chloroplastic-like [Solanum lycopersicum]	gi|460390676|ref|XM_004240906.1|	PREDICTED: Solanum lycopersicum plastidial pyruvate kinase 4, chloroplastic-like (LOC101257775), mRNA
CL8820.Contig1	2011	PREDICTED: pyruvate kinase, cytosolic isozyme-like [Solanum lycopersicum]	gi|73811194|gb|DQ114474.1|	Capsicum annuum pyruvate kinase mRNA, complete cds
CL8820.Contig2	2186	PREDICTED: pyruvate kinase, cytosolic isozyme-	gi|73811194|gb|DQ114	Capsicum annuum pyruvate kinase mRNA, complete cds

geneID	Gene Length	Nr-annotation	Nt-ID	Nt-annotation
		like [Solanum lycopersicum]	474.1|	
CL8820.Contig3	1986	PREDICTED: pyruvate kinase, cytosolic isozyme-like [Solanum lycopersicum]	gi|73811194|gb|DQ114474.1|	Capsicum annuum pyruvate kinase mRNA, complete cds
CL8820.Contig4	2024	PREDICTED: pyruvate kinase, cytosolic isozyme-like [Solanum lycopersicum]	gi|73811194|gb|DQ114474.1|	Capsicum annuum pyruvate kinase mRNA, complete cds
CL8820.Contig5	493	pyruvate kinase, cytosolic isozyme [Glycine max]	gi|264129|gb|S53332.1|	Solanum tuberosum cytosolic pyruvate kinase (PKc) gene, complete cds
Unigene12367	2029	PREDICTED: pyruvate kinase isozyme G, chloroplastic-like [Solanum lycopersicum]	gi|460400710|ref|XM_004245828.1|	PREDICTED: Solanum lycopersicum pyruvate kinase isozyme G, chloroplastic-like (LOC101263236), mRNA
Unigene16049	573	pyruvate kinase [Capsicum annuum]	gi|73811194|gb|DQ114474.1|	Capsicum annuum pyruvate kinase mRNA, complete cds
Unigene16429	2393	PREDICTED: pyruvate kinase isozyme A, chloroplastic-like [Solanum lycopersicum]	gi|460404293|ref|XM_004247570.1|	PREDICTED: Solanum lycopersicum pyruvate kinase isozyme A, chloroplastic-like (LOC101261160), mRNA
Unigene1803	2512	PREDICTED: plastidial pyruvate kinase 2-like [Solanum lycopersicum]	gi|460376853|ref|XM_004234163.1|	PREDICTED: Solanum lycopersicum plastidial pyruvate kinase 2-like (LOC101262183), mRNA
Unigene19656	730	PREDICTED: pyruvate kinase, cytosolic isozyme-like [Solanum lycopersicum]	gi|460402153|ref|XM_004246531.1|	PREDICTED: Solanum lycopersicum pyruvate kinase, cytosolic isozyme-like, transcript variant 2 (LOC101248036), mRNA
Unigene33404	536	PREDICTED: pyruvate kinase, cytosolic isozyme-like [Solanum lycopersicum]	gi|225315614|dbj|AK321766.1|	Solanum lycopersicum cDNA, clone: LEFL1029BD10, HTC in leaf
male sterility				
CL10138.Contig1	2170	PREDICTED: PHD finger protein MALE STERILITY 1-like [Solanum lycopersicum]	gi|460382355|ref|XM_004236857.1|	PREDICTED: Solanum lycopersicum PHD finger protein MALE STERILITY 1-like (LOC101257358), mRNA
CL10138.Contig2	502	PREDICTED: PHD finger protein MALE STERILITY 1-like [Solanum lycopersicum]	gi|460382355|ref|XM_004236857.1|	PREDICTED: Solanum lycopersicum PHD finger protein MALE STERILITY 1-like (LOC101257358), mRNA
CL1641.Contig1	493	male sterility protein 2, partial [Lycium barbarum]	gi|377823843|gb|JQ341412.1|	Lycium barbarum cultivar Ningqi No. 1 male sterility protein 2 (ms2) mRNA, partial cds
CL1641.Contig3	326	male sterility protein 2, partial [Lycium barbarum]	gi|377823843|gb|JQ341412.1|	Lycium barbarum cultivar Ningqi No. 1 male sterility protein 2 (ms2) mRNA, partial cds

geneID	Gene Length	Nr-annotation	Nt-ID	Nt-annotation
Unigene18482	510	cytoplasmic male sterility-associated protein [Capsicum annuum]	gi|310645225|gb|HQ232799.1|	Capsicum annuum cultivar 9704A cytoplasmic male sterility-associated protein mRNA, complete cds; mitochondrial
Unigene22461	402	male sterility protein 2, partial [Lycium barbarum]	gi|377823843|gb|JQ341412.1|	Lycium barbarum cultivar Ningqi No. 1 male sterility protein 2 (ms2) mRNA, partial cds
