# Supplementary material for: Orf165 is associated with cytoplasmic male sterility in pepper
Source: Genet Mol Biol. 2021 Sep 22;44(3):e20210030. doi: 10.1590/1678-4685-GMB-2021-0030 (PMC8459829; doi:10.1590/1678-4685-GMB-2021-0030)
Supplement: Table S5 ‒ [file 1415-4757-GMB-44-3-e20210030-s12.pdf]

Genetics and Molecular Biology

https://doi.org/10.1590/1678-4685-GMB-2021-0030

Supplementary Material to "*Orf165* is associated with cytoplasmic male sterility in Pepper"

Table S5 - DEGs involved in energy metabolism in A2 vs B2 comparison.

geneID	geneLength	B2_rawfragments(18881297)	A2_rawfragments(17590083)	B2_FPKM	A2_FPKM	log2 Ratio(A2/B2)	Up-Down-Regulation(A2/B2)	P-value	FDR
atp2									
Unigene13850	212	3	9	0.7495	2.4135	1.687126	Up	0.070067	0.130265
atp4									
Unigene11535	503	1203	1167	126.6677	131.897	0.058363	Up	0.324528	0.446896
Unigene25039	422	556	824	69.7799	111.0061	0.669756	Up	1.26E-17	2.15E-16
Unigene25237	1084	3366	3897	164.4572	204.3776	0.313525	Up	2.23E-20	4.42E-19
atp7									
Unigene30340	474	24	35	2.6816	4.1978	0.646539	Up	0.08971	0.159821
atp6									
Unigene24614	627	54	66	4.5614	5.9842	0.391682	Up	0.138552	0.229212
atp9									
Unigene14245	346	16	17	2.4491	2.7932	0.189667	Up	0.704612	0.778956
coxii									
CL1985.Contig4	466	9	0	1.0229	0	-9.99845	Down	0.002766	0.008296
Unigene26984	591	0	5	0	0.481	8.909893	Up	0.025172	0.055599
Unigene32293	600	3	0	0.2648	0	-8.04876	Down	0.143664	0.234786
coxiii									
Unigene20200	358	12	20	1.7753	3.176	0.839148	Up	0.109599	0.187611
nad1									
Unigene10713	345	13	14	1.9957	2.307	0.209123	Up	0.70581	0.777223
nad2									
CL5464.Contig1	2360	2131	92	47.8233	2.2162	-4.43155	Down	0	0
CL5464.Contig2	1861	20	531	0.5692	16.2211	4.832792	Up	########	########
nad7									
CL11266.Contig1	1026	26	33	1.3421	1.8285	0.446168	Up	0.238524	0.351778
CL1540.Contig2	3066	163	161	2.8157	2.9853	0.084382	Up	0.597492	0.692932
cob									
CL1985.Contig1	818	11	0	0.7122	0	-9.47614	Down	0.000741	0.002606
aconitase									
CL11665.Contig1	6561	2063	3912	16.6532	33.897	1.025358	Up	########	########
CL11665.Contig2	2559	215	331	4.4498	7.3534	0.724671	Up	6.48E-09	5.47E-08
CL11665.Contig3	3731	36	35	0.511	0.5333	0.061624	Up	0.854156	0.904197
Unigene25633	290	4	36	0.7305	7.0573	3.27216	Up	3.08E-08	2.40E-07

geneID	geneLength	B2_rawfragments(18881297)	A2_rawfragments(17590083)	B2_FPKM	A2_FPKM	log2 Ratio(A2/B2)	Up-Down-Regulation(A2/B2)	P-value	FDR
GAPDH									
CL4065.Contig1	519	309	495	31.5326	54.2213	0.782016	Up	3.30E-14	4.53E-13
Succinate dehydrogenase									
CL1348.Contig10	2564	3	0	0.062	0	-5.9542	Down	0.143664	0.235109
CL1348.Contig3	2798	3	0	0.0568	0	-5.82782	Down	0.143664	0.234851
CL1348.Contig4	2688	2	0	0.0394	0	-5.30012	Down	0.277504	0.395133
CL1348.Contig5	2774	3	0	0.0573	0	-5.84046	Down	0.143664	0.235008
CL1348.Contig6	1876	2	0	0.0565	0	-5.82018	Down	0.277504	0.395576
CL1348.Contig8	2422	2	0	0.0437	0	-5.44956	Down	0.277504	0.395701
CL1348.Contig9	2478	2	0	0.0427	0	-5.41616	Down	0.277504	0.39579
CL1348.Contig12	2544	6	14	0.1249	0.3129	1.324928	Up	0.054514	0.105781
CL1348.Contig14	2219	2	0	0.0477	0	-5.57592	Down	0.277504	0.395867
CL1348.Contig15	2279	2	0	0.0465	0	-5.53916	Down	0.277504	0.395387
CL1348.Contig17	2439	2	0	0.0434	0	-5.43962	Down	0.277504	0.395375
CL367.Contig1	443	46	57	5.4995	7.3148	0.411518	Up	0.149705	0.242631
CL367.Contig2	1338	1833	1979	72.5562	84.0856	0.21276	Up	5.32E-06	2.95E-05
Unigene12480	828	151	171	9.6586	11.7408	0.281645	Up	0.080156	0.146118
Unigene13723	313	21	1	3.5534	0.1816	-4.29036	Down	1.19E-05	6.21E-05
Unigene24327	2604	3381	3872	68.7658	84.533	0.297823	Up	1.59E-18	2.87E-17
Unigene20200	358	12	20	1.7753	3.176	0.839148	Up	0.109599	0.187611
mdh									
Unigene20397	616	618	827	53.1344	76.3233	0.522477	Up	7.35E-12	8.36E-11
Unigene25224	1460	6024	8240	218.5246	320.8533	0.554118	Up	########	########
Unigene25419	1754	1615	1803	48.7653	58.4384	0.261062	Up	1.25E-07	8.93E-07
pyruvate kinase									
CL6833.Contig1	1933	4084	5232	111.8979	153.875	0.459576	Up	5.31E-53	3.01E-51
CL6833.Contig2	352	41	53	6.1689	8.5598	0.472564	Up	0.114686	0.195317
CL6833.Contig3	1495	1765	2502	62.5276	95.1433	0.605609	Up	3.11E-42	1.34E-40
CL6857.Contig4	3543	788	884	11.7794	14.1845	0.268049	Up	0.000147	0.000614
CL8820.Contig1	2011	88	169	2.3176	4.7776	1.043654	Up	1.61E-08	1.30E-07
CL8820.Contig2	2186	75	132	1.8171	3.4329	0.91779	Up	7.31E-06	3.97E-05
CL8820.Contig3	1986	30	94	0.8	2.6908	1.749963	Up	4.19E-10	4.02E-09
CL8820.Contig4	2024	23	55	0.6018	1.5448	1.360064	Up	7.49E-05	0.000333
CL8820.Contig5	493	641	764	68.8619	88.1005	0.355444	Up	4.03E-06	2.29E-05
Unigene12367	2029	510	556	13.3124	15.5785	0.226786	Up	0.010306	0.026031
Unigene16429	2393	2520	3558	55.7733	84.527	0.599838	Up	2.49E-58	1.56E-56
Unigene1803	2512	4640	5598	97.8288	126.6909	0.372982	Up	5.84E-39	2.34E-37
