# Supplementary material for: Orf165 is associated with cytoplasmic male sterility in pepper
Source: Genet Mol Biol. 2021 Sep 22;44(3):e20210030. doi: 10.1590/1678-4685-GMB-2021-0030 (PMC8459829; doi:10.1590/1678-4685-GMB-2021-0030)
Supplement: Table S6 ‒ [file 1415-4757-GMB-44-3-e20210030-s13.pdf]

Genetics and Molecular Biology

https://doi.org/10.1590/1678-4685-GMB-2021-0030

Supplementary Material to *"Orf165* is associated with cytoplasmic male sterility in Pepper"

Table S6 - DEGs involved in energy metabolism in B1 vs B2 comparison.

geneID	Gene Length	B1_rawfragments (18059056)	B2_rawfragments (18881297)	B1_FPKM	B2_FPKM	log2 Ratio (B2/B1)	Up-Down-Regulation (B2/B1)	P-value	FDR
ATP1									
Unigene17921	455	16	21	1.9472	2.4444	0.328079	Up	0.501248	0.645246
ATP2									
Unigene13850	212	14	3	3.6568	0.7495	-2.28658	Down	0.005697	0.021588
ATP4									
Unigene11535	503	1058	1203	116.4723	126.6677	0.121062	Up	0.046449	0.116012
ATP7									
Unigene1469	1248	4270	4913	189.4603	208.4973	0.138133	Up	4.65E-06	4.07E-05
Unigene30340	474	16	24	1.8692	2.6816	0.520673	Up	0.267928	0.418138
ATP6									
CL5535.Contig1	533	51	59	5.2984	5.8626	0.145984	Up	0.600178	0.723048
CL5535.Contig2	404	40	67	5.4826	8.7834	0.679919	Up	0.017345	0.053413
Unigene13749	367	15	16	2.2632	2.309	0.028904	Up	0.960592	0.976344
Unigene24614	627	145	54	12.8058	4.5614	-1.48925	Down	6.80E-12	1.24E-10
Unigene32029	817	44	58	2.9822	3.7599	0.334317	Up	0.24834	0.398686
Unigene32030	1842	12	13	0.3607	0.3738	0.051467	Up	0.935528	0.968625
Unigene32031	1046	21	26	1.1117	1.3165	0.24394	Up	0.57127	0.702381
ATP9									
Unigene14245	346	8	16	1.2803	2.4491	0.93577	Up	0.134398	0.257402
ATPA									
CL6700.Contig3	5885	500	545	4.7047	4.9048	0.060092	Up	0.501898	0.645812
CL9996.Contig1	3665	976	1371	14.7462	19.8122	0.426046	Up	1.29E-12	2.50E-11
CL9996.Contig2	4420	2478	3196	31.0445	38.2959	0.302852	Up	3.57E-15	8.39E-14
COXII									

geneID	Gene Length	B1_rawfragments (18059056)	B2_rawfragments (18881297)	B1_FPKM	B2_FPKM	log2 Ratio (B2/B1)	Up-Down-Regulation (B2/B1)	P-value	FDR
Unigene32293	600	7	3	0.646	0.2648	-1.28663	Down	0.199132	0.341668
COXIII									
CL420.Contig1	860	233	521	15.0025	32.0854	1.096714	Up	1.37E-23	5.10E-22
CL420.Contig2	905	296	586	18.1112	34.2939	0.92107	Up	3.77E-20	1.20E-18
Unigene20200	358	20	12	3.0935	1.7753	-0.80118	Down	0.127472	0.245735
NAD1									
Unigene23825	673	18	44	1.481	3.4626	1.225284	Up	0.001655	0.007657
NAD2									
CL5464.Contig1	2360	1246	2131	29.2355	47.8233	0.709992	Up	1.02E-44	7.50E-43
CL8822.Contig1	2464	1	8	0.0225	0.172	2.934412	Up	0.025715	0.073262
NAD3									
CL10232.Contig1	599	5	13	0.4622	1.1494	1.314292	Up	0.078675	0.17216
CL8634.Contig1	691	4	7	0.3205	0.5365	0.743254	Up	0.432162	0.589952
NAD7									
CL1540.Contig1	1467	32	34	1.2079	1.2275	0.023222	Up	0.951222	0.97679
CL1540.Contig2	3066	90	163	1.6255	2.8157	0.79261	Up	2.05E-05	0.000159
Unigene11959	360	135	174	20.7652	25.5985	0.301892	Up	0.067891	0.153276
Unigene28657	909	608	675	37.0378	39.3286	0.08658	Up	0.283494	0.43798
NAD9									
Unigene14262	348	4	9	0.6365	1.3697	1.105628	Up	0.208304	0.352319
COB									
CL11399.Contig1	537	1	4	0.1031	0.3945	1.935981	Up	0.24032	0.389204
CL11399.Contig2	1885	207	618	6.0808	17.3638	1.51375	Up	1.58E-44	1.16E-42
CL1985.Contig1	818	9	11	0.6092	0.7122	0.225366	Up	0.74006	0.829246
aconitase									
CL11665.Contig2	2559	467	215	10.1054	4.4498	-1.18331	Down	5.05E-25	2.01E-23
CL11665.Contig3	3731	26	36	0.3859	0.511	0.405096	Up	0.278426	0.431074
CL11665.Contig4	3570	1092	1537	16.9379	22.802	0.428905	Up	4.05E-14	8.79E-13
CL7127.Contig3	875	7	8	0.443	0.4842	0.128296	Up	0.874254	0.932526
CL7127.Contig4	1085	31	54	1.5821	2.6359	0.736455	Up	0.022187	0.064817
Unigene25633	290	28	4	5.3464	0.7305	-2.87161	Down	6.18E-06	5.29E-05
Succinate dehydrogenase									
CL1348.Contig10	2564	0	3	0	0.062	5.954196	Up	0.136506	0.259986
CL1348.Contig1	2674	1	6	0.0207	0.1188	2.520832	Up	0.080607	0.175037

geneID	Gene Length	B1_rawfragments (18059056)	B2_rawfragments (18881297)	B1_FPKM	B2_FPKM	log2 Ratio (B2/B1)	Up-Down-Regulation (B2/B1)	P-value	FDR
CL1348.Contig2	1694	7	4	0.2288	0.1251	-0.87101	Down	0.3461	0.507899
CL1348.Contig3	2798	0	3	0	0.0568	5.827819	Up	0.136506	0.259701
CL1348.Contig4	2688	0	2	0	0.0394	5.300124	Up	0.267068	0.417755
CL1348.Contig5	2774	0	3	0	0.0573	5.840463	Up	0.136506	0.259898
CL1348.Contig6	1876	0	2	0	0.0565	5.820179	Up	0.267068	0.418257
CL1348.Contig7	2036	4	5	0.1088	0.1301	0.257942	Up	0.809256	0.878461
CL1348.Contig8	2422	0	2	0	0.0437	5.449561	Up	0.267068	0.418346
CL1348.Contig9	2478	1	2	0.0223	0.0427	0.937192	Up	0.658754	0.770583
CL1348.Contig12	2544	12	6	0.2612	0.1249	-1.06438	Down	0.139074	0.262726
CL1348.Contig14	2219	1	2	0.025	0.0477	0.932061	Up	0.658754	0.770735
CL1348.Contig15	2279	0	2	0	0.0465	5.539159	Up	0.267068	0.41805
CL1348.Contig17	2439	0	2	0	0.0434	5.439623	Up	0.267068	0.418037
CL367.Contig1	443	42	46	5.2499	5.4995	0.067011	Up	0.831236	0.898041
CL367.Contig2	1338	1686	1833	69.7761	72.5562	0.056366	Up	0.247098	0.396826
CL420.Contig1	860	233	521	15.0025	32.0854	1.096714	Up	1.37E-23	5.10E-22
CL420.Contig2	905	296	586	18.1112	34.2939	0.92107	Up	3.77E-20	1.20E-18
CL6700.Contig1	2460	10	23	0.2251	0.4952	1.137445	Up	0.033736	0.089186
CL6700.Contig4	4142	69	120	0.9225	1.5344	0.734054	Up	0.000636	0.003339
Unigene12355	966	1164	1523	66.7238	83.5009	0.32359	Up	7.37E-09	9.79E-08
Unigene12480	828	110	151	7.3564	9.6586	0.392814	Up	0.029439	0.080927
Unigene13723	313	11	21	1.946	3.5534	0.868688	Up	0.104789	0.21517
Unigene20200	358	20	12	3.0935	1.7753	-0.80118	Down	0.127472	0.245735
Unigene31173	241	6	10	1.3786	2.1976	0.672725	Up	0.380598	0.540422
pyruvate kinase									
CL6833.Contig2	352	96	41	15.102	6.1689	-1.29165	Down	5.64E-07	5.76E-06
CL8820.Contig3	1986	78	30	2.1748	0.8	-1.44281	Down	9.44E-07	9.30E-06
CL8820.Contig4	2024	45	23	1.2311	0.6018	-1.03259	Down	0.004337	0.017245
Unigene16049	573	377	446	36.4327	41.2238	0.178244	Up	0.077371	0.170656
Unigene33404	536	874	1524	90.2925	150.5873	0.737922	Up	1.30E-34	7.21E-33
