# Supplementary material for: Orf165 is associated with cytoplasmic male sterility in pepper
Source: Genet Mol Biol. 2021 Sep 22;44(3):e20210030. doi: 10.1590/1678-4685-GMB-2021-0030 (PMC8459829; doi:10.1590/1678-4685-GMB-2021-0030)
Supplement: Table S7 ‒ [file 1415-4757-GMB-44-3-e20210030-s14.pdf]

Genetics and Molecular Biology

https://doi.org/10.1590/1678-4685-GMB-2021-0030

Supplementary Material to *"Orf165* is associated with cytoplasmic male sterility in Pepper"

Table S7 - DEGs involved in energy metabolism in A1 vs A2 comparison.

geneID	Gene Length	B1_rawfragments (18059056)	B2_rawfragments (18881297)	B1_FPKM	B2_FPKM	log2 Ratio (B2/B1)	Up-Down-Regulation (B2/B1)	P-value	FDR
ATP1									
Unigene17921	455	9	16	1.1446	1.9991	0.804507	Up	0.183115	0.406214
ATP4									
Unigene11535	503	1027	1167	118.1509	131.897	0.158781	Up	0.01006	0.053797
Unigene25039	422	704	824	96.5372	111.0061	0.201482	Up	0.006456	0.038193
Unigene25237	1084	3192	3897	170.3994	204.3776	0.262317	Up	2.28E-14	8.08E-13
CL11262.Contig1	948	1401	1948	85.5193	116.8188	0.449951	Up	3.26E-19	1.53E-17
ATP5									
CL11262.Contig1	948	1401	1948	85.5193	116.8188	0.449951	Up	3.26E-19	1.53E-17
ATP7									
Unigene1469	1248	4177	4460	193.6798	203.1666	0.06899	Up	0.026344	0.109339
Unigene30340	474	6	35	0.7325	4.1978	2.518733	Up	3.70E-06	5.54E-05
ATP6									
CL5535.Contig1	533	41	45	4.4513	4.7997	0.108717	Up	0.729664	0.862177
CL5535.Contig2	404	26	30	3.7241	4.2216	0.180898	Up	0.644064	0.809103
Unigene13749	367	9	4	1.4191	0.6196	-1.19557	Down	0.168969	0.386416
Unigene32029	817	17	33	1.2041	2.2963	0.931356	Up	0.02834	0.115299
ATP9									
Unigene14245	346	9	17	1.5052	2.7932	0.891964	Up	0.133626	0.330096
COXII									
Unigene26984	591	1	5	0.0979	0.481	2.296656	Up	0.130923	0.327078
Unigene2101	901	575	593	36.9298	37.4164	0.018885	Up	0.823388	0.913166
COXIII									
CL420.Contig1	860	125	167	8.411	11.0395	0.392326	Up	0.021136	0.0937
CL420.Contig2	905	163	254	10.4225	15.9558	0.61438	Up	1.81E-05	0.000237
Unigene20200	358	17	20	2.7479	3.176	0.208881	Up	0.666602	0.8253
NAD1									

geneID	Gene Length	B1_rawfragments (18059056)	B2_rawfragments (18881297)	B1_FPKM	B2_FPKM	log2 Ratio (B2/B1)	Up-Down-Regulation (B2/B1)	P-value	FDR
Unigene10713	345	13	14	2.1805	2.307	0.081359	Up	0.887812	0.953924
Unigene23825	673	30	35	2.5795	2.9565	0.196799	Up	0.587348	0.775085
NAD2									
CL1003.Contig14	2225	47	20	1.2224	0.511	-1.25832	Down	0.000704	0.006113
CL8822.Contig2	3283	122	130	2.1504	2.2512	0.066089	Up	0.717606	0.852801
NAD3									
CL8634.Contig1	691	0	1	0	0.0823	6.362821	Up	0.508906	0.71947
NAD7									
CL11266.Contig1	1026	27	33	1.5228	1.8285	0.263934	Up	0.484998	0.712786
CL1540.Contig2	3066	157	161	2.9632	2.9853	0.01072	Up	0.947854	0.998748
aconitase									
CL11665.Contig1	6561	3806	3912	33.5686	33.897	0.014045	Up	0.669078	0.827004
CL11665.Contig3	3731	17	35	0.2637	0.5333	1.01605	Up	0.015142	0.07303
CL7127.Contig3	875	14	7	0.9259	0.4548	-1.02562	Down	0.123316	0.317475
Unigene25633	290	30	36	5.9863	7.0573	0.237452	Up	0.5094	0.713439
AGPase									
CL2979.Contig1	1135	27	29	1.3766	1.4526	0.077528	Up	0.84359	0.924049
GAPDH									
CL4065.Contig1	519	325	495	36.2368	54.2213	0.581404	Up	1.19E-08	2.54E-07
Succinate dehydrogenase									
CL1348.Contig10	2564	2	0	0.0451	0	-5.49506	Down	0.243408	0.481821
CL1348.Contig3	2798	3	0	0.062	0	-5.9542	Down	0.120625	0.312425
CL1348.Contig4	2688	2	0	0.0431	0	-5.42962	Down	0.243408	0.481871
CL1348.Contig5	2774	3	0	0.0626	0	-5.96809	Down	0.120625	0.312776
CL1348.Contig6	1876	1	0	0.0308	0	-4.94486	Down	0.491172	0.712857
CL1348.Contig8	2422	2	0	0.0478	0	-5.57894	Down	0.243408	0.482972
CL1348.Contig9	2478	2	0	0.0467	0	-5.54535	Down	0.243408	0.483053
CL1348.Contig11	2588	12	5	0.2683	0.1098	-1.28897	Down	0.088948	0.25568
CL1348.Contig13	1225	1	0	0.0472	0	-5.56071	Down	0.491172	0.71324
CL1348.Contig14	2219	2	0	0.0522	0	-5.70598	Down	0.243408	0.483194
CL1348.Contig15	2279	2	0	0.0508	0	-5.66676	Down	0.243408	0.482416
CL1348.Contig16	1169	1	0	0.0495	0	-5.62936	Down	0.491172	0.710897

geneID	Gene Length	B1_rawfragments (18059056)	B2_rawfragments (18881297)	B1_FPKM	B2_FPKM	log2 Ratio (B2/B1)	Up-Down-Regulation (B2/B1)	P-value	FDR
CL1348.Contig17	2439	3	0	0.0712	0	-6.15381	Down	0.120625	0.313136
CL1348.Contig18	1141	2	0	0.1014	0	-6.66391	Down	0.243408	0.483032
CL367.Contig1	443	38	57	4.9638	7.3148	0.559373	Up	0.063302	0.203736
CL367.Contig2	1338	1549	1979	66.993	84.0856	0.327848	Up	1.80E-11	5.11E-10
CL420.Contig1	860	125	167	8.411	11.0395	0.392326	Up	0.021136	0.0937
CL420.Contig2	905	163	254	10.4225	15.9558	0.61438	Up	1.81E-05	0.000237
CL6700.Contig1	2460	8	3	0.1882	0.0693	-1.44134	Down	0.137612	0.335445
Unigene12480	828	148	171	10.3435	11.7408	0.182806	Up	0.259624	0.493224
Unigene13723	313	4	1	0.7395	0.1816	-2.02579	Down	0.210546	0.442771
Unigene20200	358	17	20	2.7479	3.176	0.208881	Up	0.666602	0.8253
Unigene24327	2604	3393	3872	75.401	84.533	0.164931	Up	1.14E-06	1.88E-05
Unigene31173	241	1	2	0.2401	0.4718	0.97454	Up	0.63836	0.803888
MDH									
Unigene20397	616	616	827	57.8675	76.3233	0.39937	Up	1.73E-07	3.20E-06
Unigene25224	1460	6535	8240	259.0163	320.8533	0.308871	Up	1.92E-38	1.83E-36
pyruvate kinase									
CL6833.Contig1	1933	4202	5232	125.7936	153.875	0.2907	Up	1.77E-22	9.85E-21
CL6833.Contig3	1495	2273	2502	87.9818	95.1433	0.112897	Up	0.006903	0.040156
CL8820.Contig1	2011	136	169	3.9135	4.7776	0.287827	Up	0.083145	0.243885
CL8820.Contig4	2024	50	55	1.4295	1.5448	0.111909	Up	0.69369	0.842604
CL8820.Contig5	493	698	764	81.93	88.1005	0.104758	Up	0.165577	0.379805
Unigene12367	2029	522	556	14.8875	15.5785	0.065455	Up	0.457026	0.690114
Unigene16049	573	323	361	32.6199	35.8166	0.134876	Up	0.222464	0.459159
Unigene16429	2393	3058	3558	73.9485	84.527	0.192891	Up	5.68E-08	1.12E-06
Unigene1803	2512	5245	5598	120.826	126.6909	0.068382	Up	0.013633	0.067752
Unigene33404	536	730	882	78.812	93.5483	0.247296	Up	0.000601	0.005342
