# Supplementary material for: Orf165 is associated with cytoplasmic male sterility in pepper
Source: Genet Mol Biol. 2021 Sep 22;44(3):e20210030. doi: 10.1590/1678-4685-GMB-2021-0030 (PMC8459829; doi:10.1590/1678-4685-GMB-2021-0030)
Supplement: Table S8 ‒ [file 1415-4757-GMB-44-3-e20210030-s15.pdf]

Genetics and Molecular Biology

https://doi.org/10.1590/1678-4685-GMB-2021-0030

Supplementary Material to "*Orf165* is associated with cytoplasmic male sterility in Pepper"

Table S8 - DEGs involved in energy metabolism in A1 vs B1 comparison.

geneID	Gene Length	B1_rawfragments (18059056)	A1_rawfragments (17280870)	B1_FPKM	A1_FPKM	log2 Ratio (A1/B1)	Up-Down-Regulation(A1/B1)	P-value	FDR
ATP1									
Unigene17921	455	16	9	1.9472	1.1446	-0.76656	Down	0.206248	0.496623
ATP4									
Unigene25039	422	728	704	95.5265	96.5372	0.015184	Up	0.841642	0.942083
Unigene25237	1084	3318	3192	169.4931	170.3994	0.007694	Up	0.82943	0.934965
Unigene28876	526	88	87	9.2641	9.5712	0.047049	Up	0.828026	0.933859
ATP7									
Unigene1469	1248	4270	4177	189.4603	193.6798	0.031778	Up	0.311374	0.608504
Unigene30340	474	16	6	1.8692	0.7325	-1.35152	Down	0.044749	0.203966
ATP6									
Unigene13749	367	15	9	2.2632	1.4191	-0.67339	Down	0.275296	0.570487
Unigene32029	817	44	17	2.9822	1.2041	-1.30842	Down	0.000929	0.0105
Unigene32030	1842	12	23	0.3607	0.7226	1.002398	Up	0.048193	0.211201
Unigene32031	1046	21	30	1.1117	1.6597	0.578155	Up	0.159018	0.429726
ATP9									
Unigene14245	346	8	9	1.2803	1.5052	0.233473	Up	0.741918	0.887912
ATPA									
CL6700.Contig3	5885	500	528	4.7047	5.1918	0.142132	Up	0.11423	0.362443
COXII									
CL1985.Contig4	466	13	0	1.5448	0	-10.5932	Down	0.000166	0.002398
Unigene26984	591	0	1	0	0.0979	6.613237	Up	0.478222	0.753302
Unigene32293	600	7	0	0.646	0	-9.33539	Down	0.0093	0.066274
COXIII									
CL420.Contig1	860	233	125	15.0025	8.411	-0.83485	Down	9.87E-08	2.73E-06
CL420.Contig2	905	296	163	18.1112	10.4225	-0.79718	Down	7.67E-09	2.46E-07

geneID	Gene Length	B1_rawfragments (18059056)	A1_rawfragments (17280870)	B1_FPKM	A1_FPKM	log2 Ratio (A1/B1)	Up-Down-Regulation(A1/B1)	P-value	FDR
NAD1									
Unigene23825	673	18	30	1.481	2.5795	0.80052	Up	0.060898	0.246857
NAD2									
CL5464.Contig1	2360	1246	94	29.2355	2.3049	-3.66495	Down	########	########
CL5464.Contig2	1861	20	673	0.5951	20.9268	5.136076	Up	########	########
CL8822.Contig1	2464	1	2	0.0225	0.047	1.062736	Up	0.592338	0.811101
CL8822.Contig2	3283	203	122	3.424	2.1504	-0.67108	Down	3.91E-05	0.000671
NAD3									
CL10232.Contig1	599	5	2	0.4622	0.1932	-1.25842	Down	0.318922	0.615659
CL8634.Contig1	691	4	0	0.3205	0	-8.32418	Down	0.069691	0.267853
NAD7									
CL1540.Contig1	1467	32	4	1.2079	0.1578	-2.93633	Down	2.05E-06	4.61E-05
CL1540.Contig2	3066	90	157	1.6255	2.9632	0.866272	Up	3.75E-06	8.04E-05
Unigene32075	1986	222	114	6.1898	3.3217	-0.89797	Down	3.15E-08	9.32E-07
NAD9									
Unigene14262	348	4	14	0.6365	2.328	1.870859	Up	0.014833	0.094389
COB									
CL11399.Contig1	537	1	1	0.1031	0.1078	0.064313	Up	0.966976	0.979644
CL11399.Contig2	1885	207	84	6.0808	2.5787	-1.23762	Down	3.94E-12	1.78E-10
CL1985.Contig1	818	9	0	0.6092	0	-9.25077	Down	0.002428	0.023153
aconitase									
CL11665.Contig1	6561	3255	3806	27.4717	33.5686	0.289166	Up	4.10E-17	2.57E-15
CL11665.Contig2	2559	467	546	10.1054	12.3469	0.289022	Up	0.001457	0.015224
CL11665.Contig4	3570	1092	1096	16.9379	17.7655	0.068823	Up	0.26443	0.561849
CL7127.Contig3	875	7	14	0.443	0.9259	1.06355	Up	0.108948	0.351802
CL7127.Contig4	1085	31	30	1.5821	1.6	0.016231	Up	0.962298	0.982553
Unigene25633	290	28	30	5.3464	5.9863	0.163097	Up	0.66727	0.850473
AGPase									
CL2979.Contig1	1135	50	27	2.4394	1.3766	-0.82542	Down	0.015214	0.096198
Succinate dehydrogenase									
CL1348.Contig10	2564	0	2	0	0.0451	5.495056	Up	0.233846	0.533045
CL1348.Contig1	2674	1	2	0.0207	0.0433	1.064736	Up	0.592338	0.810306
CL1348.Contig3	2798	0	3	0	0.062	5.954196	Up	0.114348	0.360588

geneID	Gene Length	B1_rawfragments (18059056)	A1_rawfragments (17280870)	B1_FPKM	A1_FPKM	log2 Ratio (A1/B1)	Up-Down-Regulation(A1/B1)	P-value	FDR
CL1348.Contig4	2688	0	2	0	0.0431	5.429616	Up	0.233846	0.533097
CL1348.Contig5	2774	0	3	0	0.0626	5.968091	Up	0.114348	0.360971
CL1348.Contig6	1876	0	1	0	0.0308	4.944858	Up	0.478222	0.750647
CL1348.Contig8	2422	0	2	0	0.0478	5.578939	Up	0.233846	0.534548
CL1348.Contig9	2478	1	2	0.0223	0.0467	1.066379	Up	0.592338	0.813472
CL1348.Contig11	2588	8	12	0.1712	0.2683	0.648164	Up	0.330218	0.629879
CL1348.Contig12	2544	12	14	0.2612	0.3185	0.286138	Up	0.617578	0.823001
CL1348.Contig13	1225	0	1	0	0.0472	5.560715	Up	0.478222	0.751008
CL1348.Contig14	2219	1	2	0.025	0.0522	1.062122	Up	0.592338	0.813684
CL1348.Contig15	2279	0	2	0	0.0508	5.666757	Up	0.233846	0.53384
CL1348.Contig16	1169	0	1	0	0.0495	5.629357	Up	0.478222	0.748542
CL1348.Contig17	2439	0	3	0	0.0712	6.153805	Up	0.114348	0.361633
CL1348.Contig18	1141	0	2	0	0.1014	6.663914	Up	0.233846	0.534638
CL182.Contig2	1072	5	2	0.2583	0.108	-1.25802	Down	0.318922	0.61815
CL420.Contig1	860	233	125	15.0025	8.411	-0.83485	Down	9.87E-08	2.73E-06
CL420.Contig2	905	296	163	18.1112	10.4225	-0.79718	Down	7.67E-09	2.46E-07
Unigene12480	828	110	148	7.3564	10.3435	0.491653	Up	0.006549	0.050518
Unigene13723	313	11	4	1.946	0.7395	-1.39589	Down	0.092661	0.320476
Unigene31173	241	6	1	1.3786	0.2401	-2.5215	Down	0.080491	0.29051
MDH									
Unigene25224	1460	6815	6535	258.4747	259.0163	0.00302	Up	0.903562	0.975876
pyruvate kinase									
CL6833.Contig2	352	96	53	15.102	8.713	-0.7935	Down	0.001092	0.012007
CL6833.Contig3	1495	1854	2273	68.671	87.9818	0.357504	Up	2.01E-15	1.14E-13
CL6857.Contig4	3543	990	1085	15.4728	17.7212	0.195742	Up	0.00201	0.01986
CL8820.Contig1	2011	110	136	3.0289	3.9135	0.369665	Up	0.045336	0.205133
CL8820.Contig2	2186	100	161	2.5331	4.262	0.750627	Up	3.47E-05	0.000603
CL8820.Contig3	1986	78	100	2.1748	2.9138	0.422019	Up	0.052329	0.223576
CL8820.Contig4	2024	45	50	1.2311	1.4295	0.215563	Up	0.467662	0.74583
CL8820.Contig5	493	724	698	81.3199	81.93	0.010783	Up	0.887372	0.97089
Unigene16429	2393	3093	3058	71.5718	73.9485	0.04713	Up	0.200136	0.491549
Unigene1803	2512	5056	5245	111.4532	120.826	0.116493	Up	4.18E-05	0.000711
Unigene19656	730	222	127	16.8397	10.0674	-0.74218	Down	2.61E-06	5.77E-05
